# OptoPI3K, genetic code expansion, and click chemistry reveal mechanisms underlying reciprocal regulation between TRPV1 and PI3K

**DOI:** 10.1101/2023.08.29.555449

**Published:** 2023-08-30

**Authors:** Duk-Su Koh, Anastasiia Stratiievska, Subhashis Jana, Shauna C. Otto, Teresa M. Swanson, Anthony Nhim, Sara Carlson, Marium Raza, Lígia Araujo Naves, Eric N. Senning, Ryan Mehl, Sharona E. Gordon

**Affiliations:** 1 University of Washington, Department of Physiology & Biophysics; 2 Department of Biochemistry and Biophysics, Oregon State University; 3 Department of Neuroscience, University of Texas at Austin

## Abstract

Receptor tyrosine kinase signaling is characterized by complex webs of interconnected pathways that regulate diverse cellular functions. The complexity of signaling is a barrier to understanding the pathways that control any particular function. In this work, we use a novel combination of approaches and a new click chemistry probe to determine the role of one pathway in regulating cell surface expression of an ion channel and a receptor tyrosine kinase. We applied an optogenetic approach to uncouple activation of the PI3K pathway from other pathways downstream of RTK activation. In this context, we used genetic code expansion to introduce a click chemistry noncanonical amino acid into the extracellular side of membrane proteins. Applying a cell-impermeant click chemistry fluorophore allowed us to visualize delivery of membrane proteins to the PM in real time. Using these approaches, we demonstrate that activation of PI3K, without activating other pathways downstream of RTK signaling, is sufficient to traffic the TRPV1 ion channels and insulin receptors to the plasma membrane.

## INTRODUCTION

Increased sensitivity to painful stimuli in injured and inflamed tissue is due to adaptations in the peripheral and central nervous systems (McMahan et al., 2013). For peripheral sensitization, G-protein coupled receptors and receptor tyrosine kinases signal through multiple pathways to produce cellular changes across multiple time scales. Nerve growth factor (NGF) is released from leukocytes, including mast cells, eosinophils, macrophages, and lymphocytes, in response to tissue injury and inflammation (Freund-Michel and Frossard, 2008; Reis et al., 2022). Paradoxically, in addition to its role increasing sensitivity to painful stimuli, NGF also is critical for wound healing (Ebadi et al., 1997; Lambiase et al., 2000; Bonini et al., 2002). Thus, strategies to block NGF-mediated pain sensitization have the unfortunate consequence of interfering with post-injury recovery.

We and others have previously shown that NGF increases trafficking of TRPV1 to the plasma membrane (PM) in sensory neurons when it binds to its receptor, tropomyosin receptor kinase A (TrkA), and TrkA recruits and activates the enzyme phosphoinositide 3-kinase (PI3K, Class IA). (Bonnington and McNaughton, 2003; Stein et al., 2006; Zhu and Oxford, 2007a). PI3K then phosphorylates the lipid phosphoinositide 4,5-bisphosphate (PI(4,5)P_2_) to make an important signal for membrane trafficking, phosphoinositide 3,4,5-trisphosphate (PI(3,4,5)P_3_), and its presence stimulates vesicle fusion with the PM via regulated exocytosis (Czech, 2000). PI3K activity has been shown to be *necessary* for NGF-induced trafficking of TRPV1 (Bonnington and McNaughton, 2003; Stein et al., 2006; Zhu and Oxford, 2007b).

Class IA PI3Ks are obligate heterodimers composed of regulatory p85 and catalytic p110 subunits (Figure 2A)(Geering et al., 2007). The p85 regulatory subunit contains two SH2 domains (N-SH2 and C-SH2) separated by an inter-SH2 domain (iSH2). The p110 catalytic subunit interacts with the entire N-SH2-iSH2-C-SH2 region of PI3K, with the N-SH2 acting as an autoinhibitory domain to inhibit catalysis. Binding of NGF to TrkA triggers TrkA to auto-phosphorylate a pair of tyrosines. Phospho-TrkA then binds to the PI3K N-SH2. This has the dual effect of recruiting PI3K to the PM (Thorpe et al., 2015; Ziemba et al., 2016) and causing a conformational change in p85 to relieve auto-inhibition of p110 by the N-SH2 (Zhang et al., 2020). Thus, PI3K bound to phospho-TrkA on the membrane phosphorylates PI(4,5)P_2_ to generate PI(3,4,5)P_3_. PI(3,4,5)P_3_ is a well-recognized signal for membrane fusion in organisms ranging from *dictyostelium* (Nichols et al., 2015) to humans (Hawkins et al., 2006; Hawkins and Stephens, 2015). Indeed, PI(3,4,5)P_3_-triggered membrane fusion mediates tumor cell motility, with PI3K acting as an oncoprotein and its corresponding phosphatase, PTEN, as a tumor suppressor (Hawkins et al., 2006).

We recently discovered reciprocal regulation between TRPV1 and PI3K. We found that the NGF-stimulated rise in the PI3K product PI(3,4,5)P_3_ probe is much greater in cells transiently transfected with TRPV1 than in control cells without TRPV1, i.e., TRPV1 enhances NGF-mediated PI3K activity (Stratiievska et al., 2018). A fragment of TRPV1 corresponding to the cytosolic ankyrin repeat domain (ARD) was also sufficient to enhance PI3K activity in response to NGF. We also previously showed that TRPV1 binds directly to Class IA PI3K (Stein et al., 2006). Using yeast 2-hybrid assays, coimmunoprecipitation from cells, and *in vitro* binding assays with recombinant protein, we demonstrated that TRPV1 interacts directly with PI3K. We localized the binding site to the ARD of TRPV1 and the N-SH2-iSH2-C-SH2 region of p85 (p85α and p85β isoforms; Figure 2A). Thus, the ARD fragment of TRPV1 interacts directly with PI3K *and* is sufficient to enhance NGF-induced PI3K activity. Based on these findings, we propose that TRPV1 regulates NGF-induced PI3K activity via this direct interaction.

Although the ARD fragment of TRPV1 does not include the membrane spanning domains and localizes to the cytoplasm, an association between the ARD and PI3K in the context of full-length TRPV1 raises the possibility that localizing PI3K to the PM in resting cells contributes to the mechanism by which TRPV1 enhances NGF-induce PI3K activity. Pre-localization of PI3K to the PM under basal conditions could accelerate and enhance PI3K activity, once PI3K was activated via binding to auto-phosphorylated TrkA receptor in the presence of NGF. Thus, it is clear that the TRPV1 ARD fragment enhances NGF-induced PI3K activity through an allosteric mechanism, and both allostery and membrane pre-localization might contribute to TRPV1 regulation of PI3K under physiological conditions. There is precedent for regulation of PI3K via membrane localization, as both weak binding to anionic phospholipids (Corbin et al., 2004) and binding to membrane-anchored Ras are believed to contribute to enhanced PI3K activity downstream of RTK activation (Buckles et al., 2017).

The properties of conventional fluorescent labels have posed a significant barrier to understanding trafficking of membrane proteins, including TRPV1. Previous studies have used large tags to label the channel protein, typically a fluorescent protein (e.g., GFP) fused to the intracellular N- or C-terminus. Fusion to fluorescent proteins carries a number of disadvantages. Fluorescent proteins are relatively dim and are susceptible to bleaching. We and others have previously shown that the intracellular termini participate in critical protein-protein interactions, some of which are involved in channel trafficking (Morenilla-Palao et al., 2004; Stein et al., 2006; Jeske et al., 2008; Camprubi-Robles et al., 2009; Jeske et al., 2009; Xing et al., 2012; Gregorio-Teruel et al., 2015; Meng et al., 2015; Mathivanan et al., 2016). The presence of large fluorescent proteins could perturb these interactions (Tsien, 1998; Montecinos-Franjola et al., 2020). TRPV1 is expressed heavily in intracellular membranes, making it nearly impossible to optically isolate just those channels on the PM (Liu et al., 2003; Gallego-Sandín et al., 2009). Fluorescent anti-TRPV1 antibodies have been used to label extracellular epitopes to distinguish these populations (e.g., (Meng et al., 2015; Nakazawa et al., 2021)), but antibodies have the same problem of large size, and we have found that antibodies that recognize extracellular regions of TRPV1 are not very specific.

Click chemistry offers a rapid, specific, and flexible method for labeling of proteins in living cells (Lang and Chin, 2014; Nikić et al., 2015; Kenry and Liu, 2019). We have previously incorporated click chemistry-compatible noncanonical amino acids (ncAAs) into amino acid positions in the cytoplasm of mammalian cells and shown we can achieve highly efficient and rapid labeling when introducing the appropriate click chemistry probe into the medium (Jang et al., 2020; Jana et al., 2023). This approach requires co-expression of the target gene with an amber stop codon (TAG) with a plasmid encoding an evolved aminoacyl tRNA synthetase that is orthogonal to mammalian cells, together with its cognate tRNA (Chin, 2014; Uttamapinant et al., 2015). An additional plasmid encoding a dominant negative form of eukaryotic elongation release factor (DN-erf) can be expressed to increase efficiency of ncAA incorporation (Schmied et al., 2014). Incorporating a click chemistry ncAA into an extracellular position in a membrane protein and then applying a membrane-impermeant click chemistry-conjugated fluorophore would allow specific labeling of protein at the surface, even for those proteins that localize to both the surface and intracellular membranes (Gregory et al., 2016; Mateos-Gil et al., 2016; Neubert et al., 2018; Ojima et al., 2021). The recent development of click chemistry ncAAs and fluorescent probes that react very rapidly (>10^4^ m^−1^s^−1^) make this an attractive approach for tracking membrane protein trafficking in real time (Peng and Hang, 2016; Row and Prescher, 2018; Meineke et al., 2020; Jana et al., 2023).

In this work, we leverage optogenetics to activation PI3K with light, genetic code expansion to incorporate a click chemistry ncAA, and a new, membrane-impermeant click chemistry-conjugated fluorophore to interrogate the mechanism by which NGF induces trafficking of TRPV1 to the PM. By isolating the PI3K pathway downstream of NGF from the other pathways coupled to TrkA (PLCγ and MAP/ERK), we demonstrate that PI3K activity is sufficient to increase TRPV1 trafficking to the PM. We apply the same approach to the insulin receptor (InsR), a receptor tyrosine kinase, to demonstrate that our new approach is of general use for different types of membrane proteins. Insulin-dependent recycling of InsR has been extensively studied (Knutson, 1991; Chen et al., 2019). Ligand-simulated InsR endocytosis is followed by either targeting to lysosomes for degradation or recycling endosomes for reinsertion into the PM. This return to the PM is not fully understood, but it is believed to involve small Rab family GTPases (Iraburu et al., 2021). Importantly, pharmacological inhibition of PI3K has been shown to inhibit InsR trafficking to the PM (Sasaoka et al., 1999). Here we show that activation of PI3K is sufficient to increase InsR trafficking to the PM. Given the importance of InsR recycling in the development of insulin resistance, understanding the role of PI3K in regulating InsR expression on the PM is critical.

## RESULTS

### PhyB/PIF can be used to uncouple PI3K from NGF/TrkA and generate PI(3,4,5)P_3_ in response to light

**Figure 1.**
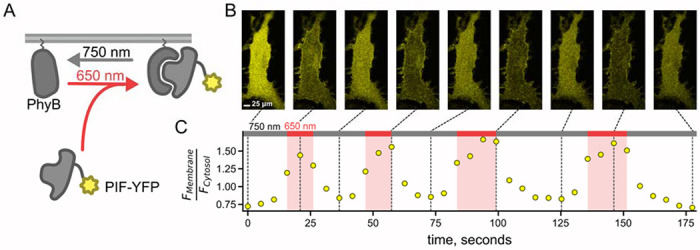
PIF-YFP translocates to PM in response to 650 nm light and back to the cytosol in response to 750 nm light. The PhyB –PIF light-inducible interaction is fully reversible on the time scale of ~10 sec and can be repeated multiple times. (A) Schematic diagram for optogenetic PhyB-PIF system. PhyB-mCherry loaded with the chromophore phycocyanobilin localizes to the PM due to a CAAX tag to induce lipidation. Illumination with 650 nm light induces a conformational change in PhyB that increases its affinity for PIF-YFP, effectively recruiting PIF-YFP to the PM. The conformational change in PhyB reverses with illumination with 750 nm light, causing PIF-YFP to dissociate and return to the cytoplasm. (B) A representative confocal experiment with an NIH3T3 cell stably expressing PhyB-mCherry-CAAX and PIF-YFP. Images were obtained at times indicated in C and are all shown on the same lookup table. During illumination with 650 nm light, PIF-YFP translocated to the PM quickly, with a corresponding decrease in cytoplasmic fluorescence. (C) Ratio of measured PIF fluorescence at region of interest (ROI) placed at the PM (F_Membrane_) and cytoplasm (F_cytosol_) from the cell in B.

Phytochrome B (PhyB) from *Arabidopsis thaliana*, together with the chromophore phycocyanobilin, undergoes a reversible conformational change in response to light (Smith, 2000; Shimizu-Sato et al., 2002). Red wavelengths (650 nm) produce a conformation of PhyB that binds to phytochrome interacting factor (PIF), whereas far-red wavelengths (750 nm) produce a conformation that releases PIF (Figure 1A). We expressed PhyB modified to include a CAAX lipidation signal, so that it localized to the PM when expressed in cultured mammalian cells (Figure 1A). We co-expressed PhyB genetically fused to mCherry with PIF fused to YFP, allowing us to select cells with high levels of PhyB and low levels of PIF (see Materials and Methods). Using confocal microscopy to image PIF-YFP in a stable cell line expressing this protein as well as PhyB-mCherry, we reproduced previously published data (Levskaya et al., 2009; Toettcher et al., 2011) to demonstrate that 650 nm light translocated PIF to the PM and 750 nm light translocated PIF to the cytoplasm (Figure 1B).

**Figure 2:**
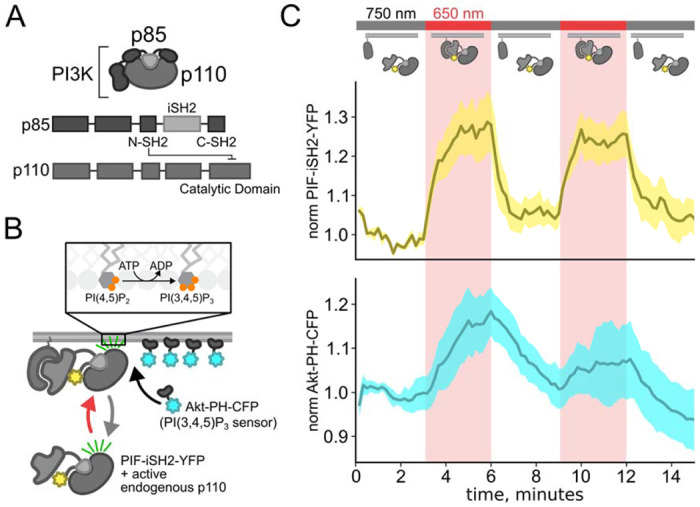
Using the PhyB/PIF system to activate PI3K with light The OptoPI3K system reversibly activates PI3K to generate PI(3,4,5)P_3_ at the PM. (A) Diagram of PI3K subunits and domains illustrating the regulatory p85 and catalytic p110 subunits. iSH2 domain in p85 subunit interacts with p110. (B) Schematic diagram for OptoPI3K system using PIF-iSH2-YFP. The iSH2 domain of p85 is fused to PIF so that translocation of PIF-iSH2-YFP, together with endogenous p110, to the PM promotes PI(3,4,5)P_3_ synthesis. (C) Monitoring PIF-iSH2-YFP translocation to and from the PM with 650 nm and 750 nm light, respectively. Synthesis of PI(3,4,5)P_3_ follows PIF-iSH2-YFP translocation to the PM, as indicated by the localization of the PI(3,4,5)P_3_ probe Akt-PH-CFP. F-11 cells transiently expressing PhyB-mCherry-CAAX, PIF-iSH2-YFP, and Akt-PH-CFP were illuminated with 750 nm or 650 nm light as indicated with the upper bar. Collected traces of PIF-iSH2-YFP (top, yellow) and Akt-PH-CFP (bottom, sky blue) normalized to the initial baselines during the first episode of 750 nm illumination. The black line indicates the mean of the data and the colored envelope represents the standard error of the mean (n=8).

It has been previously shown that the inter-SH2 domain (iSH2) of the p85 regulatory subunit of PI3K (Figure 2A) associates with the endogenous p110 catalytic subunit of PI3K (Klippel et al., 1993). Because this heterodimer is missing the autoinhibitory domains of p85, it is constitutively active, so that PI3K phosphorylates its substrate, PI(4,5)P_2_ to make PI(3,4,5)P_3_ whenever the PIF-iSH2-YFP is driven to the membrane (Figure 2B) (Suh et al., 2006; Idevall-Hagren et al., 2012). The iSH2 domain has been previously expressed as a fusion protein with PIF and has been shown to reversibly translocate to the PM and activate PI3K in response to light (Levskaya et al., 2009; Toettcher et al., 2011). Using a CFP-labelled pleckstrin homology domain from the enzyme Akt (Akt-PH-CFP), which specifically binds the PI(3,4,5)P_3_ product of PI3K (Lemmon, 2008), we confirmed that the PhyB/PIF-iSH2 machinery could be used to rapidly generate PI(3,4,5)P_3_ in response to 650 nm light (Figure 2C). In response to 750 nm light, PIF-iSH2-YFP was released from the membrane and the levels of PI(3,4,5)P_3_ returned to baseline, presumably due to the activity of the endogenous PI(3,4,5)P_3_ phosphatase PTEN. Because of the very low density of PI(3,4,5)P_3_ present in the PM even in light- or NGF-stimulated cells (Auger et al., 1989), we used total internal reflection fluorescence (TIRF) microscopy to measure PI(3,4,5)P_3_ density instead of confocal microscopy. TIRF illumination decreases exponentially with distance from the coverslip, selectively illuminating and exciting fluorophores within ~150 nm of the PM (Lakowicz, 2006; Mattheyses and Axelrod, 2006).

### Activation of PI3K, without other pathways downstream of NGF/TrkA, is sufficient to drive trafficking of TRPV1 to the PM

**Figure 3.**
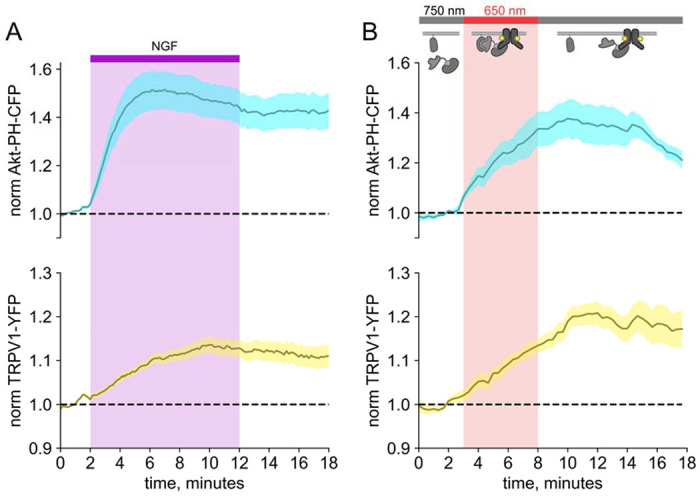
Activation of PI3K with light is sufficient to induce trafficking of TRPV1 to the PM. Simultaneous TIRF measurement of PI(3,4,5)P_3_ (cyan) and TRPV1 (yellow) in the PM in response to either (A) NGF or (B) light. (A) F-11 cells were transfected with TrkA/p75NTR, Akt-PH-CFP, and TRPV1-YFP. NGF (100 ng/mL) was applied during the times indicated by the bar/shading. Plotted are the PM-associated fluorescence in Akt-PH-CFP (top, cyan) and TRPV1-YFP (bottom, yellow) within the cell foot prints. Data are reproduced from (Stratiievska et al., 2018) (B) F-11 cells transfected with PhyB-mCherry-CAAX, PIF-ISH2 (without a fluorescent tag), Akt-PH-CFP, and TRPV1-YFP were illuminated with 750 nm or 650 nm light as indicated. Color scheme as in (B), with line indicating the mean and envelope indicated the standard error of the mean (n=13 for Akt-PH-CFP; n=16 for TRPV1-YFP). Note the poor or irreversible increase of PM PI(3,4,5)P_3_ in the PM. Inset cartoons depict the model for retention of iSH2 at the PM via binding to TRPV1.

We and others have previously demonstrated that activation of the RTK TrkA by nerve growth factor leads to increased trafficking of the ion channel TRPV1 to the PM (Zhang et al., 2005; Stein et al., 2006; Zhu and Oxford, 2007a). This increase in surface expression is associated with increased sensitivity to painful stimuli sensed by TRPV1. As shown in Figure 3A, the NGF-induced trafficking of TRPV1 follows the increase in PM PI(3,4,5)P_3_ levels. Although TrkA couples with PLCγ and the MAP/ERK pathways in addition to PI3K, significant evidence indicates that PI3K activation is *necessary* to NGF-induced trafficking of TRPV1 to the PM. A specific inhibitor of PI3K prevents NGF-stimulated sensitization (Bonnington and McNaughton, 2003; Stein et al., 2006; Stratiievska et al., 2018), a TrkA mutation that decouples it from PI3K (but not PLCγ) prevents NGF-stimulated TRPV1 trafficking (Zhang et al., 2005), and isolated sensory neurons from mice in which one isoform of a regulatory subunit of PI3K has been knocked out show decreased sensitization at the cellular level and decreased inflammatory hyperalgesia at the level of the whole organism (Zhang et al., 2005).

We used PhyB with PIF-iSH2 to test the hypothesis that PI3K activity is *sufficient* to drive trafficking of TRPV1 to the PM. Together with PhyB, PIF-iSH2 (without a fluorescent tag) and Akt-PH-CFP, we expressed TRPV1 fused to YFP. Stimulation of cells with 650 nm light gave the expected rise in PM-associated Akt-PH-CFP fluorescence and, importantly, a rise in PM-associated TRPV1-YFP fluorescence (Figure 3B). The light-induced rise in PM PI(3,4,5)P_3_ and TRPV1 qualitatively resembled those induced by NGF. Because PLCγ and the MAP/ERK were not directly activated by the 650 nm light, we conclude that activation of the PI3K pathway is sufficient to cause TRPV1 trafficking to the PM.

### TRPV1 expression appears to trap PI3K at the PM

We have previously shown that TRPV1 potentiates receptor-activated PI3K activity, increasing PI(3,4,5)P_3_ levels in NGF-stimulated cells above those observed in control cells that did not express TRPV1 (Stratiievska et al., 2018). Expression of an isolated fragment of TRPV1 corresponding to the amino-terminal ankyrin repeat domain (ARD) was sufficient to potentiate NGF-induced PI3K activity (Stratiievska et al., 2018), and the ARD bound the p85 regulatory subunit of PI3K in yeast 2-hybrid experiments, in co-immunoprecipitation experiments using cell lines and acutely isolated dorsal root ganglion neurons, and *in vitro* experiments using recombinant, purified protein (Stein et al., 2006). Our interpretation is that the TRPV1 allosterically regulates PI3K via a direct interaction of the ARD with p85.

When we monitored PIF-iSH2 localization in light-activated PI3K experiments, we found that expression of TRPV1 trapped PIF-iSH2 at the PM upon stimulation with 650 nm light, so that it no longer translocated to the cytoplasm in response to 750 nm light (Figure 3B and Figure 3 – figure supplement 1A). The 650 nm light-induced increase in plasma-membrane associated Akt-PH-CFP was similarly irreversible in TRPV1-expressing cells (Figure 3B and Figure 3 – figure supplement 1A). A version of PIF-YFP that did not include the iSH2 domain reversibly translocated to the PM in response to light (Figure 3 – figure supplement 2), indicating that localization to the membrane required both the iSH2 fragment of PI3K and TRPV1. Related TRPM4 ion channels, which do not include an ankyrin repeat domain, did not trap PIF-iSH2 at the PM (Figure 3 – figure supplement 1B), indicating that this effect is specific to TRPV1.

Given the direct interaction between TRPV1 and the p85 subunit of PI3K, we propose that when the PIF-iSH2/p110 form of PI3K is translocated to the PM in response to 650 nm light, it may encounter TRPV1, bind, and become trapped. These data are consistent with a mechanism for TRPV1-mediated potentiation of receptor-activated PI3K activity that involves localization of PI3K at the PM (see Discussion).

### Resolving changes in surface expression requires improved approaches

TIRF microscopy was needed to resolve the NGF- and light-induced increase in PM PI(3,4,5)P_3_ levels but is a poor approach for distinguishing membrane protein localization in the PM from that in the endoplasmic reticulum and other intracellular compartments (Stratiievska et al., 2018). The TIRF evanescent field gives illumination that decreases exponentially with distance from the coverslip: The ~5 nm adjacent to the coverslip that includes the PM is indeed more strongly illuminated than any 5 nm slice in the interior of the cell. Nonetheless, integrating the fluorescence interior to this first 5 nm slice demonstrates that 90% of the light intensity falls on regions more intracellular than the PM (Ambrose, 1961). For membrane proteins with very high endoplasmic reticulum localization, like TRPV1 (Tominaga et al., 1998), this makes any changes in fluorescence intensity measured with TIRF an underestimate of true changes in surface localization (Stratiievska et al., 2018).

**Figure 4.**
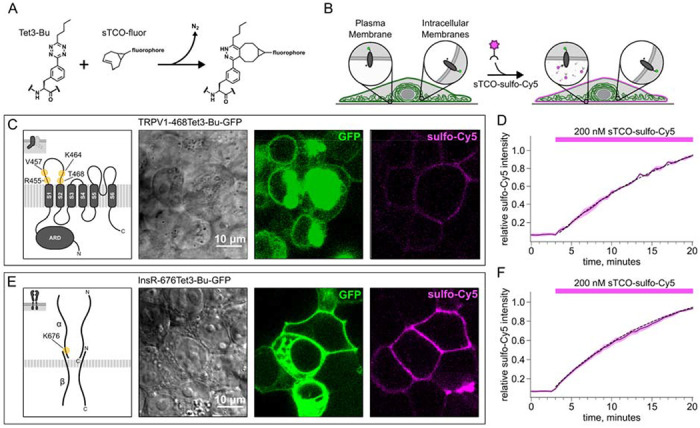
Labeling the TRPV1 and InsR with membrane-impermeant sTCO-Cy5. Confocal imaging illustrates the labeling of membrane proteins incorporating the ncAA Tet3-Bu with sTCO-sulfo-Cy5 in HEK293T/17 cells. The membrane impermeable dye labeled only the proteins on the PM. (A) Schematic of the reaction between Tet3-Bu and sTCO-conjugated dyes. (B) Cartoon representing the selective labeling of membrane proteins incorporating Tet3-Bu at an extracellular site with membrane-impermeant sTCO-sulfo-Cy5. (C & E) Confocal images of HEK293T/17 cells expressing (C) TRPV1-468Tet3-Bu-GFP or (E) InsR-676Tet3-Bu-GFP. GFP fluorescence reflects expression of the proteins in the confocal volume across the field of view. Initially the cells did not show any detectable Cy5 fluorescence but after incubation of several minutes of 200 nM sTCO-sulfo-Cy5 showed Cy5 fluorescence at the PM. The Cy 5 images shown for (C) TRPV1-Tet3-Bu and (E) InsR-Tet3-Bu were obtained at the end of the experiment (20 minutes). For better visibility, sulfo-Cy5 is shown as inverted black-white images. (D & F) The graphs summarize the Cy5 fluorescence at the PM in (D) TRPV1-Tet3-Bu-GFP or (F) InsR-Tet3-Bu-GFP expressing cells. Solid traces represent the mean and envelopes the standard error of the mean (n=3 for TRPV1 and n=11 for InsR). Dashed traces represent a fit to the mean with a single exponential (tau = 17.8 seconds for TRPV1; tau = 13.8 seconds for InsR). Fits to the individual time courses for all the cells gave a mean of 18.2 seconds for TRPV1 (± 2.2 seconds) and 19.3 seconds for InsR (±4.0 seconds).

**Figure 5:**
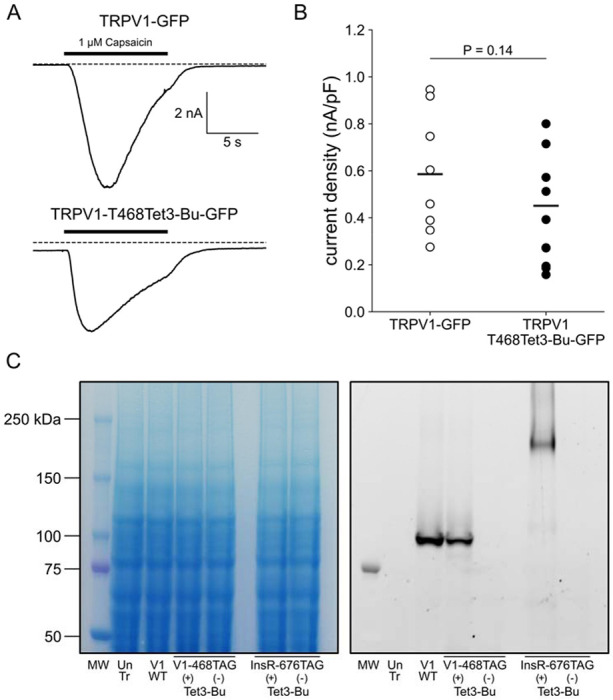
Incorporation of Tet3but into TRPV1 and InsR. (A) Whole-cell patch clamp recording demonstrates that TRPV1 incorporating Tet3-Bu remains functional. Application of 1 uM capsaicin to HEK-293T/17 cells transfected with either TRPV1-GFP (top) or TRPV1-468Tet3-Bu (bottom) induced inward currents at a holding potential of −60 mV. For the Tet3-Bu-incorporating TRPV1 channels, cells were co-transfected with a second plasmid encoding the aminoacyl tRNA synthetase for incorporating Tet3-Bu and the corresponding tRNA, and a third plasmid encoding a dominant negative form of elongation release factor (see Materials and Methods). (B) Collected data from all cells with peak capsaicin-activated currents normalized to cell areas. (C) In-gel fluorescence screening demonstrates expression of full-length TRPV1-468Tet3-Bu or InsR-676Tet3-Bu requires the presence of Tet3-Bu (‘Tet3 (+)’). Wild type TRPV1-GFP is shown for reference. Detergent-extracted cell lysates were run on SDS/PAGE. GFP fluorescence in the gels was measured and then cells were stained with Coomassie blue dye.

To circumvent the issues with TIRF microscopy, we implemented an inverse-electron-demand Diels–Alder cycloaddition click chemistry approach to selectively label PM localized proteins (Arsic et al., 2022). For this application genetic code expansion is used to incorporate a noncanonical amino acid at an extracellular site (Neubert et al., 2018; Bessa-Neto et al., 2021; Kuhlemann et al., 2021). We used Tet3-Bu, which can be efficiently labelled with a cyclopropane-fused trans-cyclooctene (sTCO; Figure 5A) with no detectable off-target reactivity within the time scale of our experiments (Figure 4 – figure supplement 1D) (Jang et al., 2020; Jana et al., 2023). Our goal was to express Tet3-Bu-incorporating membrane proteins on the surface and measure labeling with a membrane-impermeant sTCO-coupled dye (Figure 4B) under control conditions and then again after activation of PI3K with either NGF or 650 nm light.

We used amber codon suppression to site-specifically incorporate Tet3-Bu (Jang et al., 2020). This involved introducing a TAG stop codon at a selected site within the coding sequence that would place the noncanonical amino acid on the extracellular side of the PM. We then co-expressed one plasmid encoding TRPV1 with a TAG codon fused to GFP, a second plasmid encoding an aminoacyl tRNA synthetase evolved to incorporate the noncanonical amino acid and the orthogonal tRNA, and a third plasmid encoding a dominant negative elongation release factor, which increased efficiency of Tet3-Bu incorporation (Schmied et al., 2014). The cell culture medium was then supplemented with Tet3-Bu.

In selecting the extracellular site for ncAA incorporation into TRPV1, we focused on the S1-S2 loop (Figure 4A) because the S3-S4 loop is very short (5 amino acids) and we wished to avoid the pore turret between S5 and S6, which has been implicated in gating of the pore (Yang et al., 2015; Bae et al., 2016; Jara-Oseguera et al., 2016; Ma et al., 2016). In our initial screen, we tested incorporation at four positions, R455, V457, K464 and T468 (Figure 4) and used a C-terminal GFP fusion to facilitate screening. We found that TRPV1-K464Tet3-Bu-GFP and TRPV1-T468Tet3-Bu-GFP produced robust, capsaicin-activated currents when interrogated with whole-cell patch-clamp (Figure 5A,B), whereas TRPV1-R455Tet3-Bu-GFP and TRPV1-V457Tet3-Bu-GFP of incorporation did not (data not shown). We focused on TRPV1-T468Tet3-Bu-GFP, as TRPV1-K464Tat3-Bu-GFP was not efficiently labelled in subsequent experiments (data not shown). We next tested whether expression of full-length TRPV1-T468Tet3-Bu-GFP required incorporation of Tet3-Bu using in-gel GFP fluorescence. As shown in Figure 5B, detergent solubilized cell lysates from cells expressing TRPV1-T468Tet3-Bu-GFP showed a band at the expected molecular weight when the medium was supplemented with Tet3-Bu, but no GFP fluorescence at this molecular weight was observed when the medium lacked Tet3-Bu. These data indicate that TRPV1-T468Tet3-Bu-GFP incorporated Tet3-Bu without measurable incorporation of natural amino acids at the TAG codon site.

In developing the method for quantifying surface expression of TRPV1 in this study, our aim was to identify an approach that would be useful for studying trafficking of any membrane protein. We therefore tested our system on the InsR, a receptor tyrosine kinase we and others have used in previous studies of ncAA incorporation (Figure 4A) (Nikić et al., 2015; Jones et al., 2021). As for TRPV1, in-gel GFP fluorescence showed that InsR-676Tet3-Bu-GFP was present in detergent solubilized cell lysates only when the medium was supplemented with Tet3-Bu (Figure 5B).

### Developing a membrane-impermeant sTCO dye

Strained trans-cyclooctenes bearing functional groups such as spin labels and fluorophores have been used previously for in-cell studies (Murrey et al., 2015; Ryan et al., 2022; Jana et al., 2023). To determine whether existing sTCO-conjugated dyes would give the required PM-selective labeling, we tested whether sTCO-TAMRA (Jang et al., 2020) or sTCO-JF646 (Jana et al., 2023) were membrane impermeant. We also synthesized and tested sTCO-fluorescein. Unfortunately, these compounds readily equilibrated across the cell PM at the concentrations required for labeling (Figure 4–figure supplement 1). We therefore synthesized a new sTCO-sulfo-Cy5 conjugate (see Figure 9, which contains two negative charges at physiological pH to minimize passive diffusion through membrane and therefore selectively label membrane proteins at the cell surface (Hoffmann et al., 2015; Nikić et al., 2015; Kozma et al., 2016; Lam et al., 2018; Keller et al., 2020).

To determine whether sTCO-sulfo-Cy5 would selectively label a membrane protein with an extracellular Tet3-Bu, we measured Cy5 fluorescence in the presence of 200 nM sTCO-sulfo- Cy5 in the bath. At this low concentration, background fluorescence due to the dye in the bath is very low. The click chemistry reaction between Tet3-Bu incorporated at the extracellular sites and sTCO-sulfo-Cy5 effectively concentrated the dye at the PM, allowing significant membrane labeling to be observed for both membrane proteins examined (TRPV1 and InsR; Figure 4). Importantly, sTCO-sulfo-Cy5 did not appear to equilibrate across the cell membrane and did not label untransfected cells (i.e., those without GFP; Figure 4 – figure supplement 1). *In vitro*, the click chemistry reaction between free Tet3-Bu ncAA and sTCO occurs with a rate of 2x10^4^ M^−1^s^−1^; this reaction rate increased by 4 fold when Tet3-Bu was incorporated on the surface of GFP (Jang et al., 2020). The low background fluorescence recorded with 200 nM dye in the bath allowed us to measure the rate of labeling for TRPV1-468Tet3-Bu-GFP and InsR-646Tet3-Bu-GFP with sTCO-sulfo-Cy5. As shown in Figure 4D, the reaction rate we measured was approximately 1x10^4^ M^−1^s^−1^ for both proteins, somewhat slower than expected for a protein in solution and on par with the reaction between the free amino acid and sTCO in solution (Meijer et al., 1998; Taylor et al., 2011).

### Click chemistry labeling can be used to measure NGF-induced trafficking of TRPV1 to the PM

**Figure 6:**
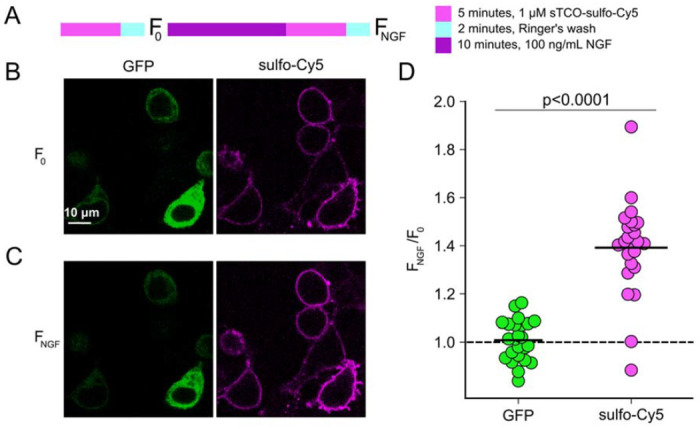
Click chemistry labeling of TRPV1-468Tet3-Bu-GFP with sTCO-Cy5 to measure NGF-induced trafficking of TRPV1 to the PM. HEK293T/17 cells expressing TRPV1-Tet3-Bu-GFP and NGF receptor were labeled with extracellular sTCO-sulfo-Cy5 and inspected with confocal microscopy. (A) Experimental protocol. Cells were incubated with 1 μM sTCO-sulfo-Cy5 for 5 min and free dye removed from the bath by washing for 2 minutes with dye-free Ringer’s solution (‘pulse-chase’ labeling. F_0_). Then the cells were treated with 100 ng/mL NGF for 10 min before the second sulfo-Cy5 labeling (F_NGF_). (B) Confocal images of HEK293T/17 cells expressing TRPV1-Tet3-Bu-GFP after initial sTCO-sulfo-Cy5 labeling. (C) Confocal images of HEK293T/17 cells expressing TRPV1-Tet3-Bu-GFP after the 10 minute treatment with NGF and subsuquent sTCO-sulfo-Cy5 labeling. (D) Summary scatter plot from multiple measurements, with individual experiments shown as dots and the mean of the experiments as black bars. The effect of NGF on GFP and sulfo-Cy5 signals is presented as a ratio of F_NGF_/F_0_.

We next asked whether click chemistry labeling could resolve trafficking of new TRPV1 channels to the PM in response to NGF. At the beginning of the experiment, we labeled surface TRPV1-468Tet3-Bu-GFP with a pulse of 1 μM sTCO-sulfo-Cy5 and then washed the label from the bath (Figure 6A). We used this higher concentration of sTCO-dye to label the PM channels more rapidly, as the NGF-induced trafficking of TRPV1 to the PM occurs with kinetics comparable to labeling with 200 nM sTCO-sulfo-Cy5. We then treated the cells with NGF for ten minutes and again pulse labelled the surface channels with a brief exposure to 1 μM sTCO-sulfo-Cy5. We observed a ~1.5 fold increase in PM Cy5 staining, indicating that NGF induced trafficking of TRPV1 to the surface (Figure 6B-D). In contrast, the total number of TRPV1 channels did not change, as evidenced by the total intensity of GFP fluorescence, which was not affected by NGF. This NGF-induced increase in surface expression is indeed greater than the ~1.1-fold increase in NGF-induced surface expression of TRPV1 observed in TIRF experiments (Figure 3).

### Click chemistry labeling can be used to measure light-induced trafficking to the PM

**Figure 7.**
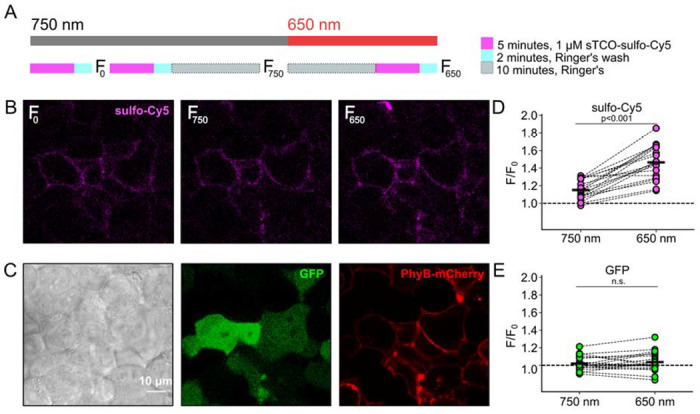
Measuring light-activated PI3K-induced TRPV1 trafficking to the PM using click chemistry (A) Illustration of the experimental protocol. HEK293T/17 cells expressing TRPV1-468Tet3-Bu-GFP and the (B) Confocal images sulfo-Cy5 obtained at different stages as depicted in (A). (C) Bright field (left) and confocal (middle and right) images obtained at the end of experiment. Comparison of bright-field (left) and GFP (middle) distinguishes TRPV1-Tet3-Bu-GFP expressing cells from untransfected cells. PhyB-mCherry images (red) indicate that most TRPV1-positive cells expressed significant levels of the PhyB/PIF machinery for activating PI3K. (D and E) Summary scatter plots from multiple experiments, with individual cells shown as dots and the mean shows as black lines. The effects of 750 and 650 nm illumination on sulfo-Cy5 (D) and GFP (E) are presented as ratios of fluorescence intensity after illumination with the indicated wavelength of illumination to the initial fluorescence.

**Figure 8.**
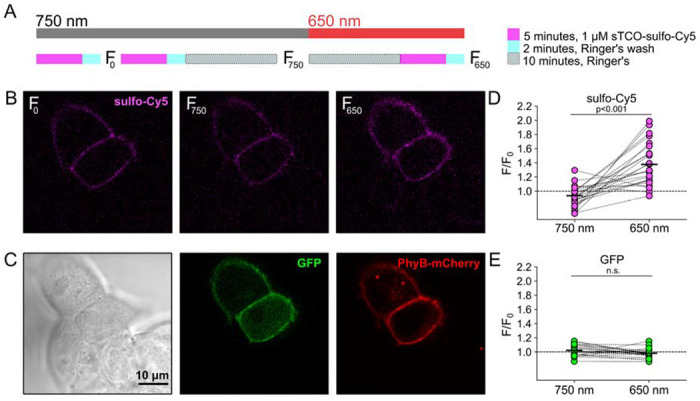
Measuring light-activated PI3K-induced InsR trafficking to the PM using click chemistry Illustration of the experimental protocol. HEK293T/17 cells expressing InsR-676Tet3-Bu-GFP and the (B) Confocal images sulfo-Cy5 obtained at different stages as depicted in (A). (C) Bright field (left) and confocal (middle and right) images obtained at the end of experiment. Comparison of bright-field (left) and GFP (middle) distinguishes InsR-676Tet3-Bu-GFP expressing cells from untransfected cells. PhyB-mCherry images (red) indicate that most TRPV1-positive cells expressed significant levels of the PhyB/PIF machinery for activating PI3K. (D and E) Summary scatter plots from multiple experiments, with individual cells shown as dots and the mean shows as black lines. The effects of 750 and 650 nm illumination on sulfo-Cy5 (D) and GFP (E) are presented as ratios of fluorescence intensity after illumination with the indicated wavelength of illumination to the initial fluorescence.

**Figure 9.**
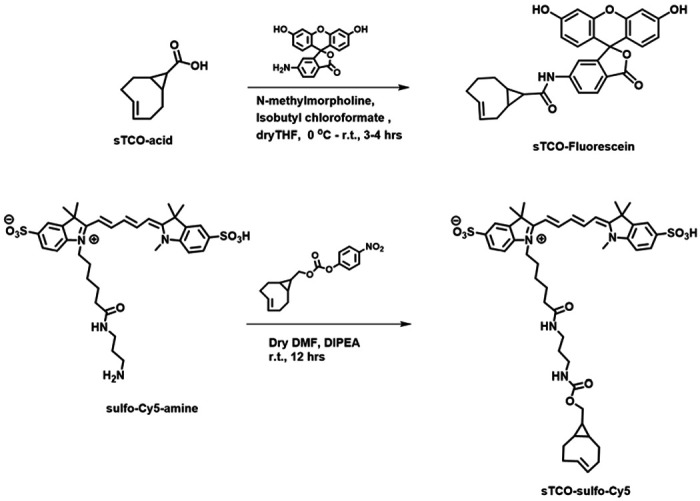
Synthesis of sTCO-Fluorescein and sTCO-sulfo-Cy5.

We next asked whether click chemistry labeling could be executed in cells in which we also used the PhyB/PIF machinery for activating PI3K. Results from these experiments with TRPV1-468Tet3-Bu-GFP and InsR-676Tet3-Bu-GFP and shown in Figure 7 and Figure 8, respectively. As an internal control, after initially labeling cells with sTCO-sulfo-Cy5, we exposed the cells to ten minutes of 750 nm light followed by a second pulse labeling with sTCO-sulfo-Cy5. The GFP fluorescence, representing total expression, did not change as a result of the 10-minute exposure to 750 nm light. As discussed below, there was a small increase in surface expression of TRPV1-468Tet3-Bu-GFP, as measured from sTCO-sulfo-Cy5 labeling under these conditions, whereas no detectable change in surface expression of InsR-676Tet3-Bu-GFP was observed. We next used a 10-minute exposure to 650 nm light to activate PI3K. For both TRPV1 and InsR, activating PI3K with light gave a significant increase in surface expression, determined from sulfo-Cy5 fluorescence, without a change in total protein levels, assayed using GFP fluorescence. For TRPV1, the amplitude of the increase in surface expression, ~1.4-fold, was similar to that observed in click chemistry experiments in response to NGF (Figure 6). The increase in surface expression of InsR-K676-Tet3-Bu was similar.

## DISCUSSION

We have previously identified reciprocal regulation between TRPV1 and PI3K. Receptor-mediated activation of PI3K increases trafficking of TRPV1 to the PM. TRPV1 expression in turn enhances receptor-mediated PI3K activity. Previously, we found that a fragment of TRPV1 corresponding to the ARD is sufficient to enhance receptor-mediated PI3K activity, indicating that at least one component of this regulation is allosteric. However, our previous data did not rule out that the direct binding of PI3K to TRPV1 (Stein et al., 2006), an integral membrane protein, might contribute to enhanced PI3K activity by localizing PI3K to the PM. Here, we show that TRPV1 traps the iSH2 fragment of PI3K at the PM, when the iSH2 domain is sent to the PM using optogenetic machinery to activate PI3K. These data are consistent with a mechanism in which both allosteric regulation and localization to the PM underlie TRPV1 regulation of PI3K activity.

Trapping of iSH2 fused to PIF at the plasma membrane by TRPV1, together with endogenous p110 that it recruits, likely explains the slightly increased sTCO-sulfo-Cy5 labelling of TRPV1 we observed after the 10-minute exposure of TRPV1-expressing cells to 750 nm light (Figure 3B and Figure 7B, D). In these experiments, cell imaging and stray light in the room appears to have been sufficient to create a “leak” of PIF-iSH2-YFP to the plasma membrane during the 750 nm light exposure. We propose that the PIF-iSH2-YFP then became trapped at the plasma membrane by TRPV1, and membrane-localized iSH2/p110 then synthesized PI(3,4,5)P_3_. In this scenario, the elevated PI(3,4,5)P_3_ levels would have induced the trafficking of TRPV1 to the plasma membrane that we observed in response to 750 nm light exposure. In contrast, no trafficking of InsR to the plasma membrane was observed as a result of 750 nm light exposure. As well, PIF-YFP, without the iSH2 domain, was not trapped at the PM by TRPV1 (Figure 3 – figure supplement 1) and TRPM4 channels, lacking ankyrin repeat domains, do not trap PIF-iSH2-YFP at the PM (Figure 3 – figure supplement 2). These data further support a model in which TRPV1 contributes to enhanced receptor-mediated PI3K activity by localizing PI3K to the plasma membrane.

Here, we present proof-of-principle experiments demonstrating the simultaneous use of an optogenetic approach for activation PI3K and genetic code expansion/click chemistry to interrogate regulation of membrane protein trafficking. This combination of approaches is especially useful for membrane proteins with significant expression in the endoplasmic reticulum and/or other intracellular compartments, which makes it very difficult to identify proteins in the PM definitively when using fusions with GFP or similar genetically encoded labels. In addition to distinguishing proteins at the surface, using a small ncAA and click chemistry is likely much less perturbing to protein structure and function than fusion with a ~20-30 kDa protein such as GFP, SNAP-tag or HaloTag. Restricting the ncAA and fluorescent label to an extracellular region/loop is also less likely to interfere with protein-protein interactions on the intracellular side, compared with a large protein fusion to an intracellular N- or C-terminal of a protein of interest.

Successful implementation of this approach required overcoming a number of barriers: efficient incorporation of Tet3-Bu into the extracellular sites; demonstration that the Tet3-Bu-incorporating protein had the expected functional properties; development of a membrane impermeant sTCO-coupled dye; and optimization of the PhyB/PIF and genetic code expansion machinery in the same cells. Incorporation of Tet3-Bu at the extracellular side of TRPV1 was well tolerated in the S1-S2 loop, with two of four sites yielding functional, capsaicin-activated channels. We have found incorporation efficiency to be difficult to predict. Screening through desirable sites typically is the best approach, with approximately one half to one third of selected sites showing reasonable levels of ncAA incorporation.

There is significant room for improvement of these methods, particularly in developing more rapid labeling of the ncAA. As shown in Figure 5, complete labeling with a low concentration of sTCO-dye required more than 10 minutes. Monitoring membrane protein trafficking to the surface in real time will require a reaction that is at least five- to 10-fold faster. Increasing the speed of labeling can be accomplished using ≥1 μM sTCO-dye, but at such high concentrations of dye the background signal from dye in solution interferes with imaging. We have recently developed new ncAAs with the tetrazine ring more proximal to the amino acid beta carbon, which we term Tet4, that react with sTCO-based labels with rates as fast as 10^6^ M^−1^s^−1^ (Jana et al., 2023). The aminoacyl tRNA synthetases that incorporate Tet4 amino acids into mammalian cells are not as efficient as those for Tet3 amino acids. Improving the efficiency of incorporating Tet4 amino acids would allow us to express Tet4-incorporating proteins and measure delivery of proteins to the PM in real time.

Another limitation to our approach is the need to co-express five to six plasmids to use PhyB/PIF and Tet/sTCO machinery simultaneously. Developing stable cell lines for ncAA incorporation would reduce the need for multiple plasmid transfection and, perhaps, lead to more uniform expression across cell populations. The PhyB/PIF machinery requires supplementing cell medium with the phycocyanobilin chromophore, making it incompatible with *in vivo* experiments. Introducing four genes, PcyA, H01, Fd, and Fnr, and knocking down knocking out biliverdin reductase A leads to efficient synthesis of phycocyanobilin in mammalian cells (Uda et al., 2017; Uda et al., 2020). It is therefore possible that genetically modified animals might one day express the PhyB/PIF machinery and chromophore and allow optogenetic activation of PI3K *in vivo*. However, there is as yet no method for synthesizing the necessary ncAAs within cells, precluding *in vivo* use of the PhyB/PIF with tetrazine-based genetic code expansion.

PI3K is a universal signal for trafficking to the PM. In *dictyostelium*, PI3K activation at the leading edge of cells underlies chemotaxis towards nutrients (Nichols et al., 2015). In macrophages, PI3K at the leading edge of cells drives motility towards the object of phagocytosis (Hawkins et al., 2006; Hawkins and Stephens, 2015). In the cases discussed here, membrane proteins constitute the cargo delivered to the PM in response to PI3K signaling. A vast literature has examined the mechanisms by which InsR is recycled from the PM in response to insulin (Knutson, 1991; Ye et al., 2017; Chen et al., 2019), but less is known about ways in which delivery of InsR to the PM is regulated. For TRPV1, PI3K was identified as an important signal that enhances surface expression in response to nerve growth factor, but the steps between PI3K activation and fusion of vesicles containing TRPV1 cargo with the PM are a mystery. The tools developed here provide a means by which the cellular mechanisms underlying PI3K-mediated delivery of membrane proteins can be interrogated.

## MATERIALS AND METHODS

### Molecular Biology

The cDNAs used in this study were provided by: rat TRPV1 in pcDNA3.1 – David Julius, UCSF, San Francisco, CA (Caterina et al., 1997); the human insulin receptor (accession AAA59452.1) with the K676TAG mutation and C-terminal GFP in a plasmid based on pEGFP– Edward Lemke, European Molecular Biology Laboratory (EMBL), Heidelberg, Germany (Nikić et al., 2015); eukaryotic elongation release factor 1 with E55D mutation in pCDNA5-FRT– Jason Chin, Medical Research Council Laboratory of Molecular Biology, Cambridge, England (Schmied et al., 2014); TrkA (rat) in the pcCMV5 vector and p75NTR (rat) in the pcDNA3 vector from Mark Bothwell, University of Washington, Seattle, WA; PH-Akt-cCerulean in pcDNA3-k vector- Orion Weiner, UCSF, San Francisco, CA (Toettcher et al., 2011) and *Methanosarcina barkeri* R2–84-RS aminoacyl tRNA synthetase/tRNA_CUA_ in the pAcBac1 plasmid – Ryan Mehl, University of Oregon, Corvallis, OR (Jang et al., 2020). Gibson cloning was used to generate the C-terminal GFP fusion for TRPV1 and to introduce 468TAG stop codon. All constructs were verified using Sangar sequencing.

### Cell Culture/transfection/solutions

The experiments in Figure 1 were performed in an NIH/3T3 cell line stably expressing PhyB-Cherry-CAAX, PIF-iSH2-YFP, and Akt-PH-CFP. These cells were a gift form Orion Weiner (UCSF, San Francisco, CA). NIH-3T3 cells were cultured at 37°C, 5% CO_2_ in Dulbecco’s modified Eagle’s medium (Invitrogen, Grand Island, NY) supplemented with 10% bovine calf serum (Hyclone, Logan, UT), L-Glutamine (Invitrogen), and penicillin/streptomycin (Lonza, Switzerland).

F-11 cells (a gift from M.C. Fishman, Massachusetts General Hospital, Boston, MA; (Francel et al., 1987)), a hybridoma of rat dorsal root ganglion neurons and mouse neuroblastoma cells, were used for experiments in Figures 2 and 3, as they are an appropriate model for studying NGF-induced signaling in pain receptor neurons. F-11 cells were incubated in Ham’s F-12 Nutrient Mixture Gibco) supplemented with 20% fetal bovine serum, penicillin/streptomycin, and HAT supplement (100 μM sodium hypoxanthine, 400 nM aminopterin, 16 μM thymidine; Invitrogen). Click chemistry experiments in Figures 4 through 8 were performed in HEK293T/17 cells (Catalog #CRL-11268, ATCC, Gaithersburg, MD) because these cells are optimized for transfection with multiple plasmids. HEK293T/17 cells were incubated according to manufacturer’s instructions in DMEM supplemented with 10% fetal bovine serum and penicillin/streptomycin.

Cells were transfected using Lipofectamine 2000 (Invitrogen) or JetPrime (Polyplus, Illkirch, France) according to the instructions of the manufacturers. Cells were passaged on to 25-mm round glass coverslips (Warner Instruments, Hamden, CT) 12 hours after transfection and cultured until used for experimentation 16–48 hours later. The coverslips were coated with poly-L-lysine to aid cell attachment (Sigma-Aldrich, St. Louis, MO .3For the optogenetic system, HEK293T/17 cells were transfected with PhyB-mCherry-CAAX (5 μg/well in 6-well culture plate), PIF-ISH2-YFP (or PIF-YFP) 0.5 μg. The 10:1 ratio helped to express more PhyB membrane target and lesser amount of PIF cargo. When cytoplasmic PIF-ISH2 was highly expressed, its translocation toward the PM upon activating 650 nm light appeared significantly reduced, likely due to the difficulty of separating signal in the cytoplasm from that at the PM. For expression of TAG-encoding cDNAs, HEK293T/17 cells in 6-well plates at 30-50% confluency were transfected target gene cDNAs at 2:1 ratio, with a total of 2 μg/well. Dominant negative elongation factor (DN-elf- E55D; 0.4 μg) was added to reduce truncation of the protein at the TAG codon. For other constructs, 1-3 μg cDNA was used for transfection.

During experiments, cells were perfused with Ringer’s solution (in mM: 140 NaCl, 4 KCl, 1 MgCl2, 1.8 CaCl2, 10 HEPES and 5 glucose, pH 7.3). All experiments were performed at room temperature.

### Optogenetic experiments

Cells were incubated in the dark with 10 μM PCB in the culture medium at for at least 1 hour prior to experiments. To avoid photodamage of Opto3K, handling of cells during and subsequent to PCB loading was performed in the dark room using a green safety flashlight (Levskaya et al., 2009). Note that PhyB loaded with PCB is extremely sensitive to light, light of any wavelength between 560 and 650 nm can activate Phytochrome B once PCB is added (Braslavsky et al., 1997; Katrin Anders, 2015). Therefore, experiments were carried out in the presence of 750 nm deactivating light and care was taken to avoid (or minimize) the exposure of cells to 560-650 nm illumination from microscopy or the external environment, including the computer monitor.

### TIRF and confocal imaging

The total internal fluorescence (TIRF) imaging set up was as described previously (Stratiievska et al., 2018). We used an inverted microscope (Nikon Ti-E) equipped for with a 60x TIRF objective (NA 1.49). For activating/deactivating opto-PI3K with light, we used HQ630/20 X (42490) and HQ760/40 X (226723) excitation filters inserted in the overhead condenser filter slider. The whole experimental chamber was illuminated with activating or deactivating light using the maximum intensity (100 W at full spectrum) of the condenser lamp. Light intensity at the focal plane was 2.4 mW and 3.7 mW for 650 nm and 750 nm illumination, respectively.

Cyan Fluorescent Protein (CFP) fusion proteins were imaged using excitation from a 440 nm laser and a 480/40 nm emission filter. Yellow Fluorescent Protein (YFP) fusion proteins were monitored using the 514 nm line of an argon laser and a 525/50 nm emission filter. mCherry was excited at 561 nm and fluorescence was collected with 605/70 nm emission filter.

Time-lapse images were obtained every 10 or 20 seconds using either a QuantEM or an Evolve EMCCD camera (Photometrics). Movies were then processed using ImageJ software (NIH) (Rasband, 1997; Schneider et al., 2012). Regions of interest (ROI) were drawn around the footprint of individual cells and the average ROI pixel intensity was measured. Background fluorescence from cell-free areas were subtracted. Measurements were analyzed using Excel (Microsoft, Redmond, WA) and Igor Pro (WaveMatrics, Portland, OR) softwares, Traces were normalized by the average intensity during 1 or 3 min time period prior to activating 650 nm light.

The confocal images were obtained with a Zeiss 710 confocal microscope (Zeiss, Oberkochen, Germany). Illumination of 650-750 nm lights were controlled as described for TIRF microscopy experiments. CFP, YFP, mCherry, and JF646 were excited with 440, 514, 561, 633 nm lasers, respectively. Data were analyzed with ImageJ. The look up table of images were changed during analysis but the same range was applied for all images within a time series experiment.

### Electrophysiology

Currents were recorded using the standard patch clamp technique (Hamill et al., 1981). Borosilicate glass electrodes had a resistance of 3–6 MΩ when filled with internal solution (in mM; 140 KCl, 5 NaCl, 0.1 EGTA, and 10 HEPES, pH 7.3). Ringer’s saline was used in the extracellular bath solution. Current through ion channels was measured in whole-cell configuration and the membrane potential was held at −60 mV. To activate TRPV1, the agonist capsaicin was applied for 10 seconds using a local perfusion system which allowed the solution to exchange within 1 second (Koh and Hille, 1997). Currents were recorded with an EPC-9 amplifier (HEKA Elektronik, Lambrecht (Pfalz), Germany) and analyzed using Igor Pro software.

### Chemical synthesis

All purchased chemicals were used without further purification. Thin-layer chromatography (TLC) was performed on silica 60F-254 plates. Flash chromatographic purification was done on silica gel 60 (230-400 mesh size). ^1^H NMR spectra were recorded at Bruker 400MHz. The chemical shifts were shown in ppm and are referenced to the residual nondeuterated solvent peak CD_3_OD (δ =3.31 in ^1^H NMR), as an internal standard. Splitting patterns of protons are designated as follows: s-singlet, d-doublet, t-triplet, q-quartet, quin-quintet, m-multiplet, bs-broad singlet.

#### sTCO-JF646:

Synthesized according to the published procedure (Jana et al., 2023).

#### sTCO-Fluorescein:

In dry tetrahydrofuran (THF) (3 mL), sTCO-CO_2_H (10 mg, 0.06 mmol) (O’Brien et al., 2018), and N-methylmorpholine (10 μL, 0.09 mmol) were taken under nitrogen atmosphere and stirrer under ice cold condition. Isobutyl chloroformate (10 μL, 0.07 mmol) was added dropwise to the reaction mixture and stirrer for 5 minutes. After that, 6-aminofluorescein (24 mg, 0.07 mmol) in dry THF (1 mL) was added portion-wise and the reaction mixture was allowed to warm to room temperature and stirring was continued for another 3 hours. The solvent was evaporated, and the residue dissolved in ethyl acetate. The solution was washed with water and saturated sodium bicarbonate solution. The organic layer was dried over sodium sulfate (Na_2_SO_4_) and the product (16 mg, 0.032 mmol) was purified by silica gel column chromatography (10-15% methanol in dichloromethane). Yield- 53%. ^1^H NMR (400MHz, CD_3_OD) *δ* 7.91 (1H, d), 7.65 (1H, d), 7.06 (1H, d), 6.89 (1H, s), 6.84-6.82 (1H, m), 6.74-6.67 (1H, m), 6.65 (1H, s), 6.59-656 (1H, m), 6.23 (1H, d), 5.92-5.86 (1H, m), 5.27-5.20 (1H, m), 2.48 (1H, d), 2.39-2.25 (3H, m), 2.04-1.94 (2H, d), 1.37-1.32 (1H, m), 1.24 (1H, t), 1.07 (1H, t), 1.01 (1H, d), 0.84-0.75 (1H, m).

#### sTCO-sulfo-Cy5:

In 1 mL of dry dimethylformamide (DMF), 5 mg of the sulfo-Cy5-amine (0.007 mmol) and 3.5 mg (0.01 mmol) of activated 4-nitrophenyl ester of sTCO (Jang et al., 2020) were added under nitrogen atmosphere. Followed by N,N-diisopropylethylamine (DIPEA) (10 μL, 3 equiv.) was added to the reaction mixture and allowed to stirrer at room temperature for 12 hours. After that, the solvent was concentrated onto silica gel under reduced pressure and the product (4 mg, 0.004 mmol) was purified by silica gel column chromatography (20-30% methanol in dichloromethane). At the beginning of column run, added 50 mL of 50% ethyl acetate and hexane to remove the DMF solvent. Yield 57%. ^1^H NMR (400MHz, CD_3_OD) *δ* 8.31 (2H, t), 7.97 (1H, s), 7.89 (2H, d), 7.87 (1H, dd), 7.33 (2H, d), 6.67 (1H, t), 6.32 (2H, dd), 5.87-5.79 (1H, m), 5.13-5.06 (1H, m), 4.13 (2H, t), 3.88 (2H, d), 3.72 (2H, quin.), 3.64 (3H, s), 3.21 (2H, q), 3.16 (2H, t), 3.09 (2H, t), 2.86 (4H, d), 2.29 (1H, d), 2.23-2.17 (3H, m), 1.89-1.79 (2H, m), 1.60 (2H, t), 1.38 (6H, s), 1.36 (6H, s), 0.91-0.83 (2H, m), 0.59-0.42 (2H, m), 0.45-0.37 (1H, m).

### In-gel fluorescence

HEK293T/17 cells were harvested 36-48 hours after transfection and treated with 1x SDS sample buffer (Invitrogen). Equal amount of proteins were loaded into 3-8% Tris-acetate gradient gels and electrophoresed at 150 V for 1 hr. Fluorescence image of the gel was obtained with an Amersham ImageQuant 800 (Cytiva, Marlborough, MA). GFP fluorescence was collected with excitation at 460 nm and a 525/20 nm emission filter). The gel was subsequently stained with Quick Coomassie solution and destained with distilled water for each 2 hrs before image acquisition.

### Statistical analysis

Data are presented as mean ± SEM, *n* is the number of single cells. Student’s *t* test was used to test the significant difference between two groups, with paired test performed as appropriate. *P* < 0.05 was regarded as significant.

## Supplementary Material

1
