# Supplementary material for: Genetic code expansion, click chemistry, and light-activated PI3K reveal details of membrane protein trafficking downstream of receptor tyrosine kinases
Source: bioRxiv. 2024 May 14:2023.08.29.555449. Originally published 2023 Aug 30. Preprint. [Version 3] doi: 10.1101/2023.08.29.555449 (PMC10491195; doi:10.1101/2023.08.29.555449)
Supplement: Supplementary file 1 [file NIHPP2023.08.29.555449v3-supplement-1.pdf]

bioRxiv preprint doi: https://doi.org/10.1101/2023.08.29.555449; this version posted May 14, 2024. The copyright holder for this preprint (which was not certified by peer review) is the author/funder, who has granted bioRxiv a license to display the preprint in perpetuity. It is made available under aCC-BY 4.0 International license.

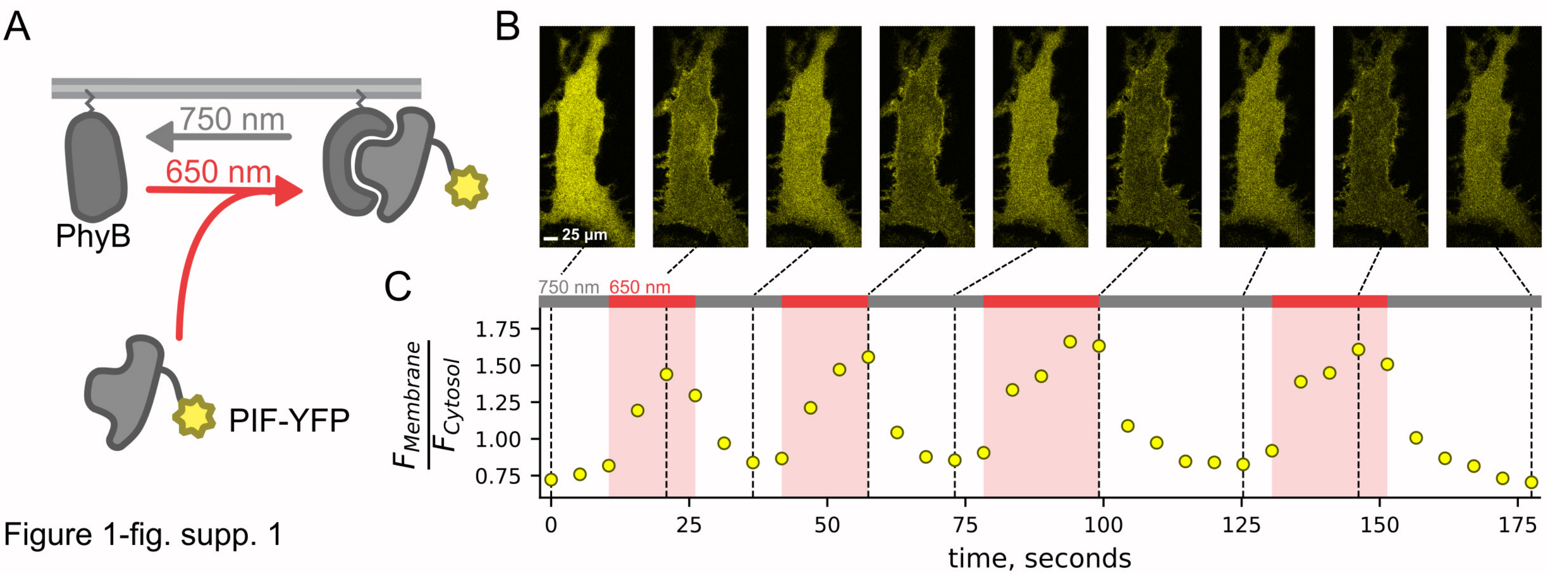

Figure 1-fig. supp. 1

bioRxiv preprint doi: https://doi.org/10.1101/2023.08.29.555449; this version posted May 14, 2024. The copyright holder for this preprint (which was not certified by peer review) is the author/funder, who has granted bioRxiv a license to display the preprint in perpetuity. It is made available under aCC-BY 4.0 International license.

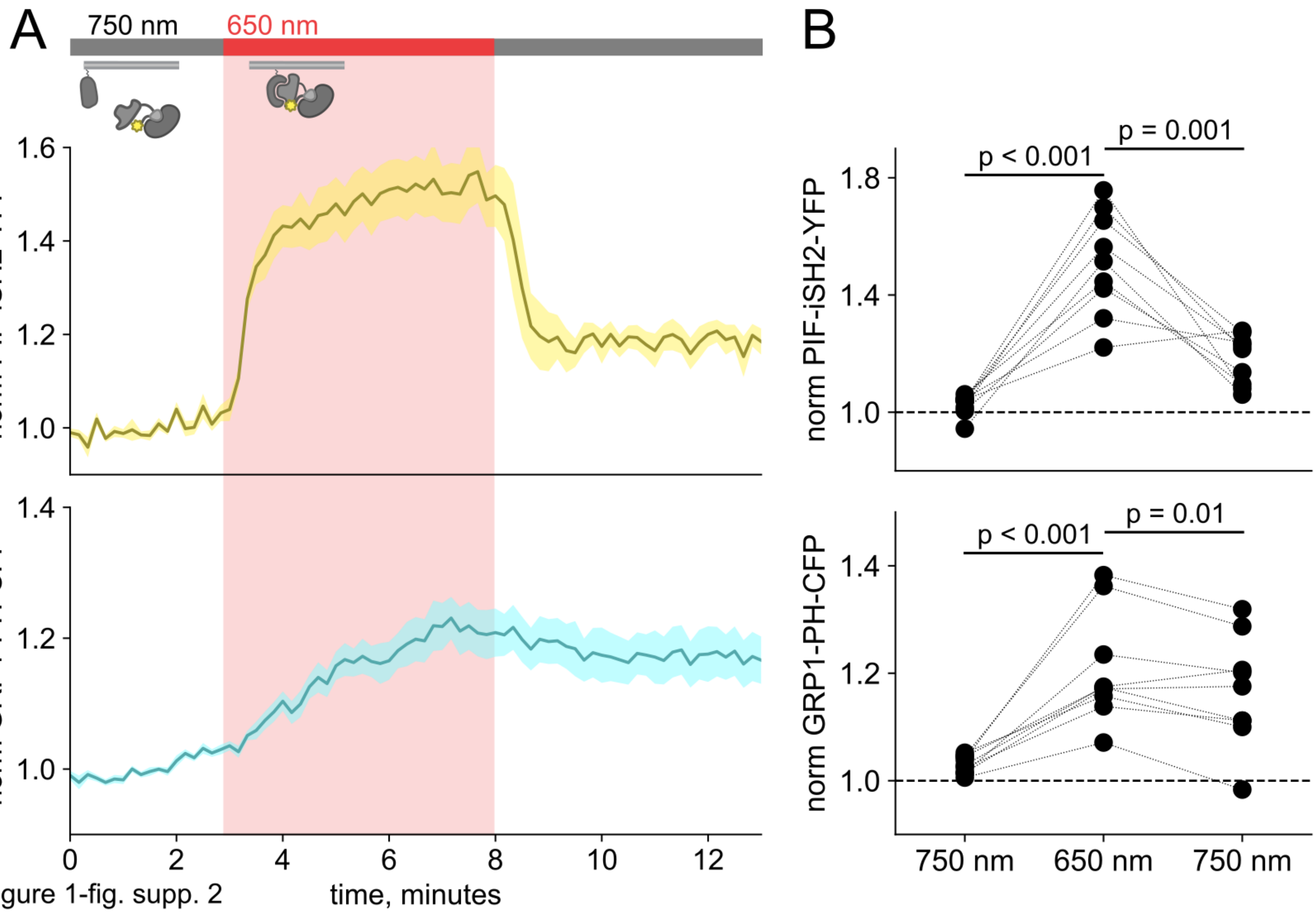

Figure 1-fig. supp. 2

bioRxiv preprint doi: https://doi.org/10.1101/2023.08.29.555449; this version posted May 14, 2024. The copyright holder for this preprint (which was not certified by peer review) is the author/funder, who has granted bioRxiv a license to display the preprint in perpetuity. It is made available under aCC-BY 4.0 International license.

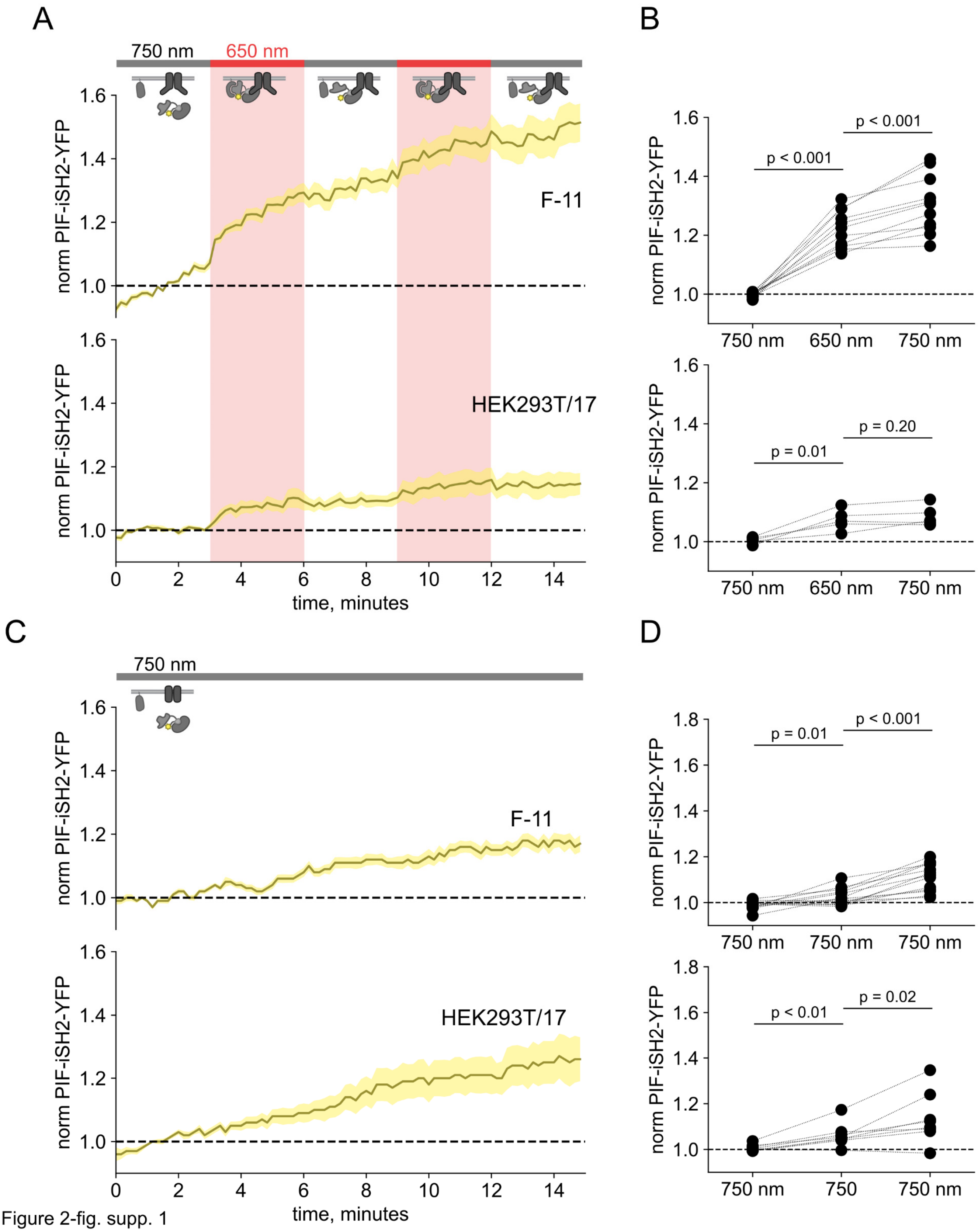

Figure 2-fig. supp. 1

bioRxiv preprint doi: https://doi.org/10.1101/2023.08.29.555449; this version posted May 14, 2024. The copyright holder for this preprint (which was not certified by peer review) is the author/funder, who has granted bioRxiv a license to display the preprint in perpetuity. It is made available under aCC-BY 4.0 International license.

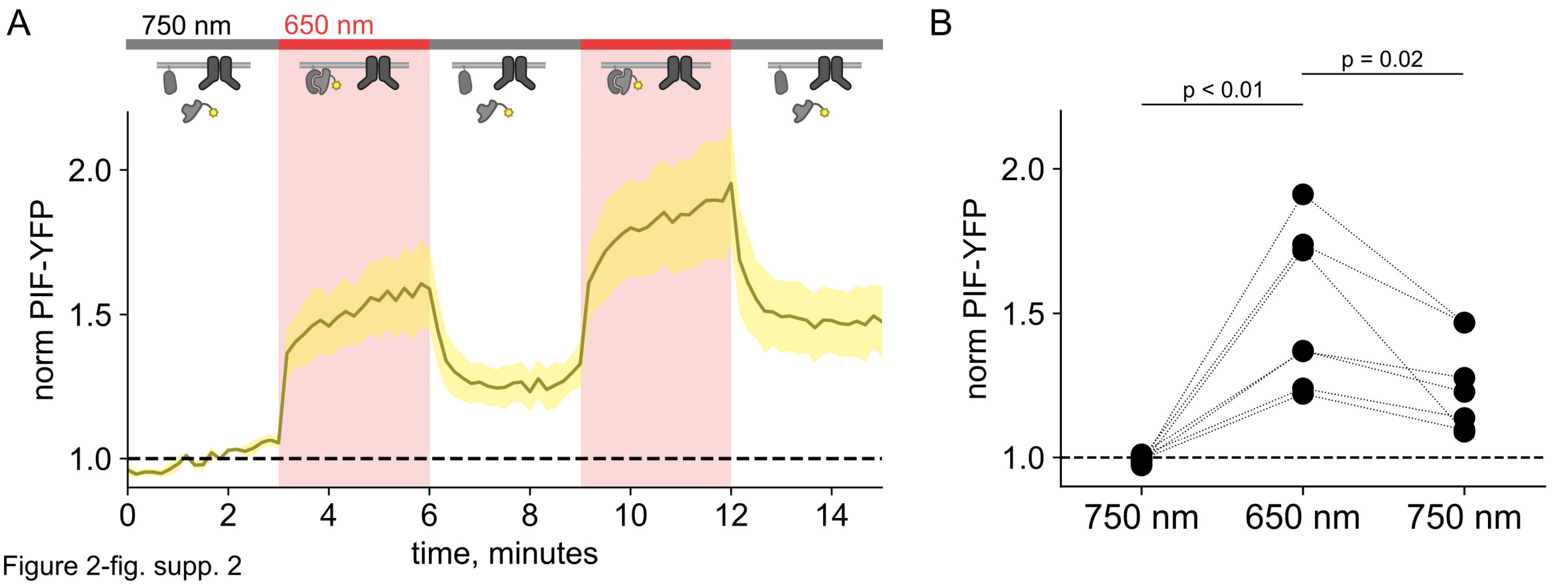

Figure 2-fig. supp. 2

bioRxiv preprint doi: https://doi.org/10.1101/2023.08.29.555449; this version posted May 14, 2024. The copyright holder for this preprint (which was not certified by peer review) is the author/funder, who has granted bioRxiv a license to display the preprint in perpetuity. It is made available under aCC-BY 4.0 International license.

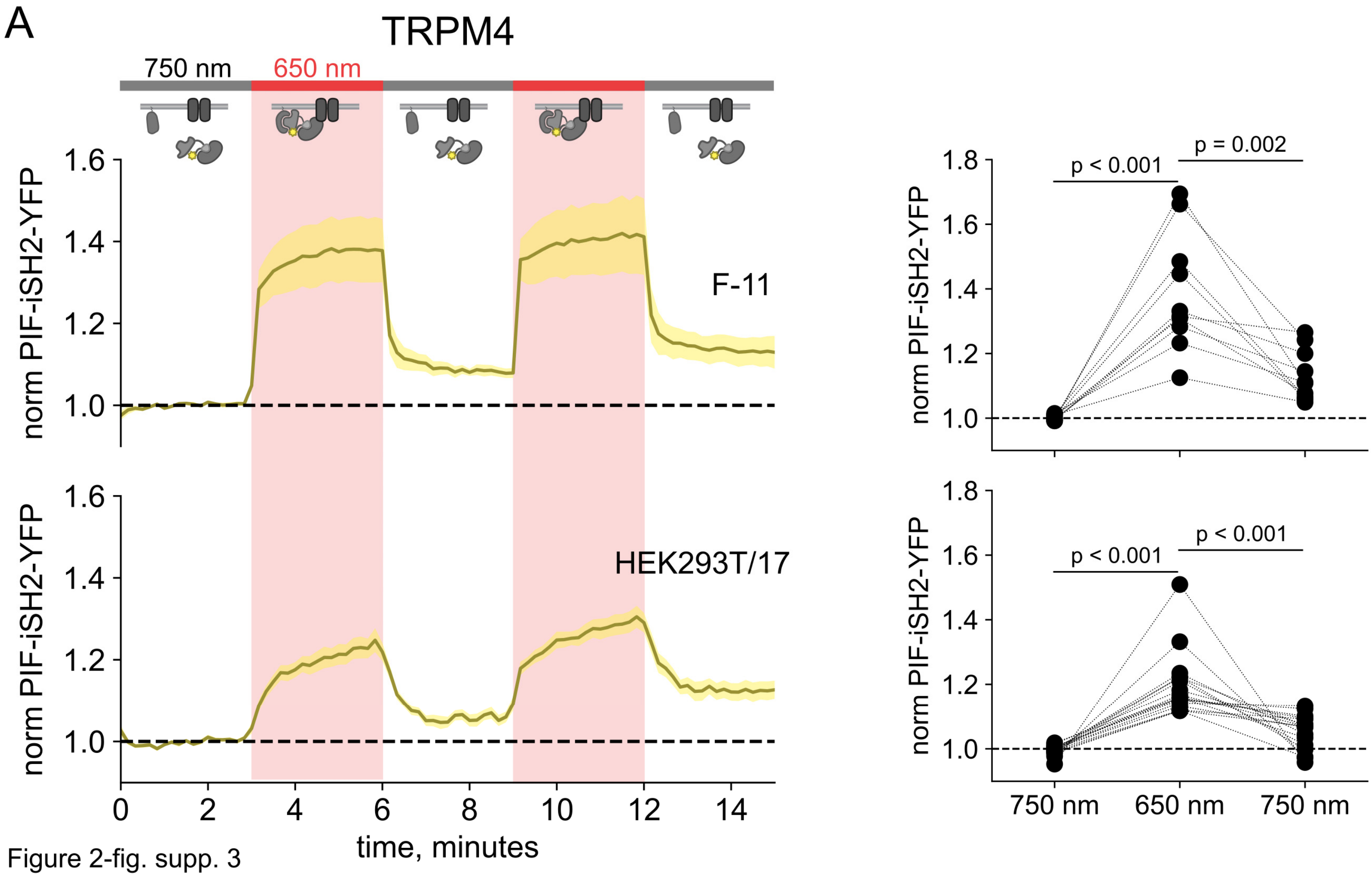

Figure 2-fig. supp. 3

bioRxiv preprint doi: https://doi.org/10.1101/2023.08.29.555449; this version posted May 14, 2024. The copyright holder for this preprint (which was not certified by peer review) is the author/funder, who has granted bioRxiv a license to display the preprint in perpetuity. It is made available under aCC-BY 4.0 International license.

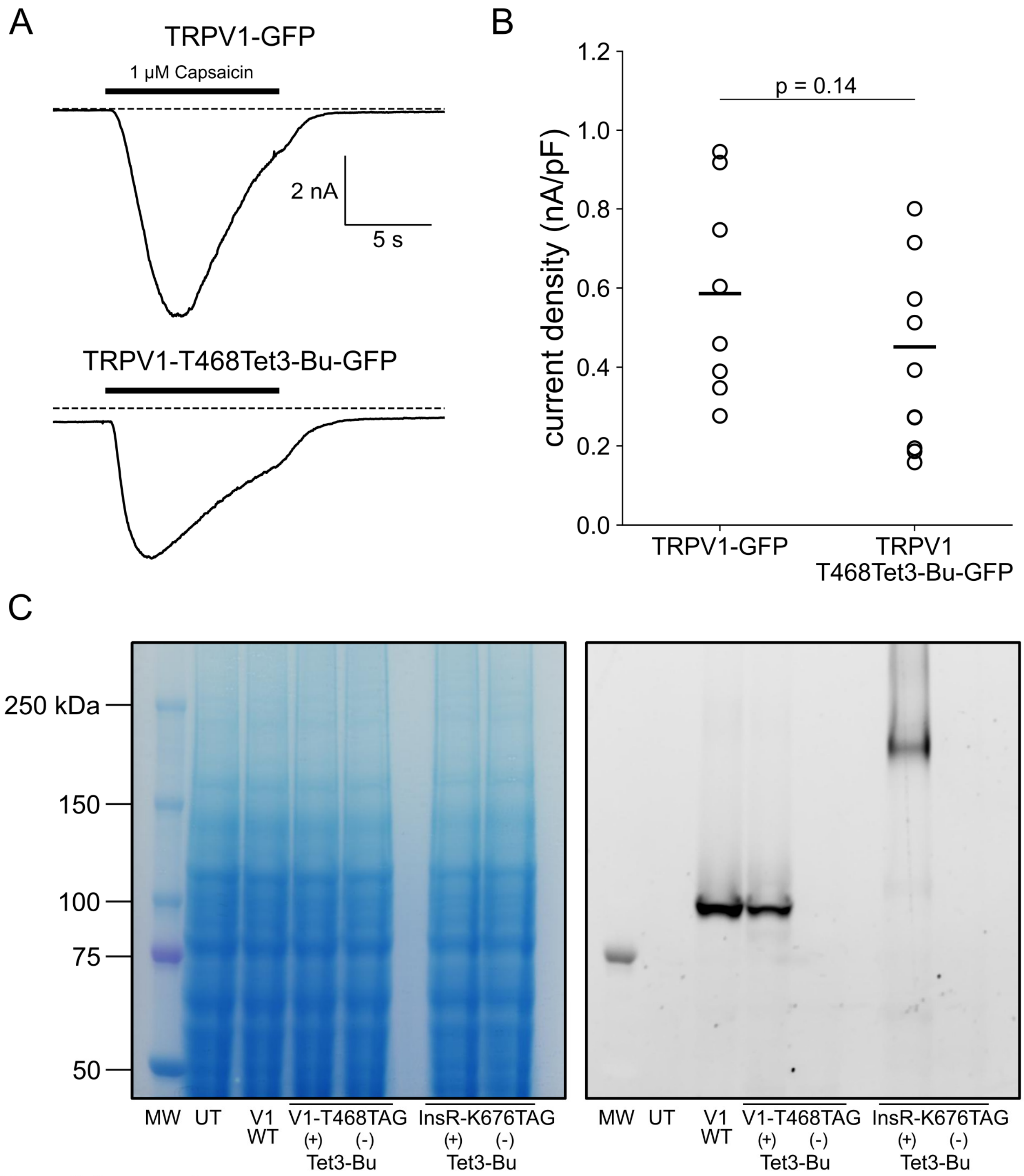

Figure 3-fig. supp. 1

bioRxiv preprint doi: https://doi.org/10.1101/2023.08.29.555449; this version posted May 14, 2024. The copyright holder for this preprint (which was not certified by peer review) is the author/funder, who has granted bioRxiv a license to display the preprint in perpetuity. It is made available under aCC-BY 4.0 International license.

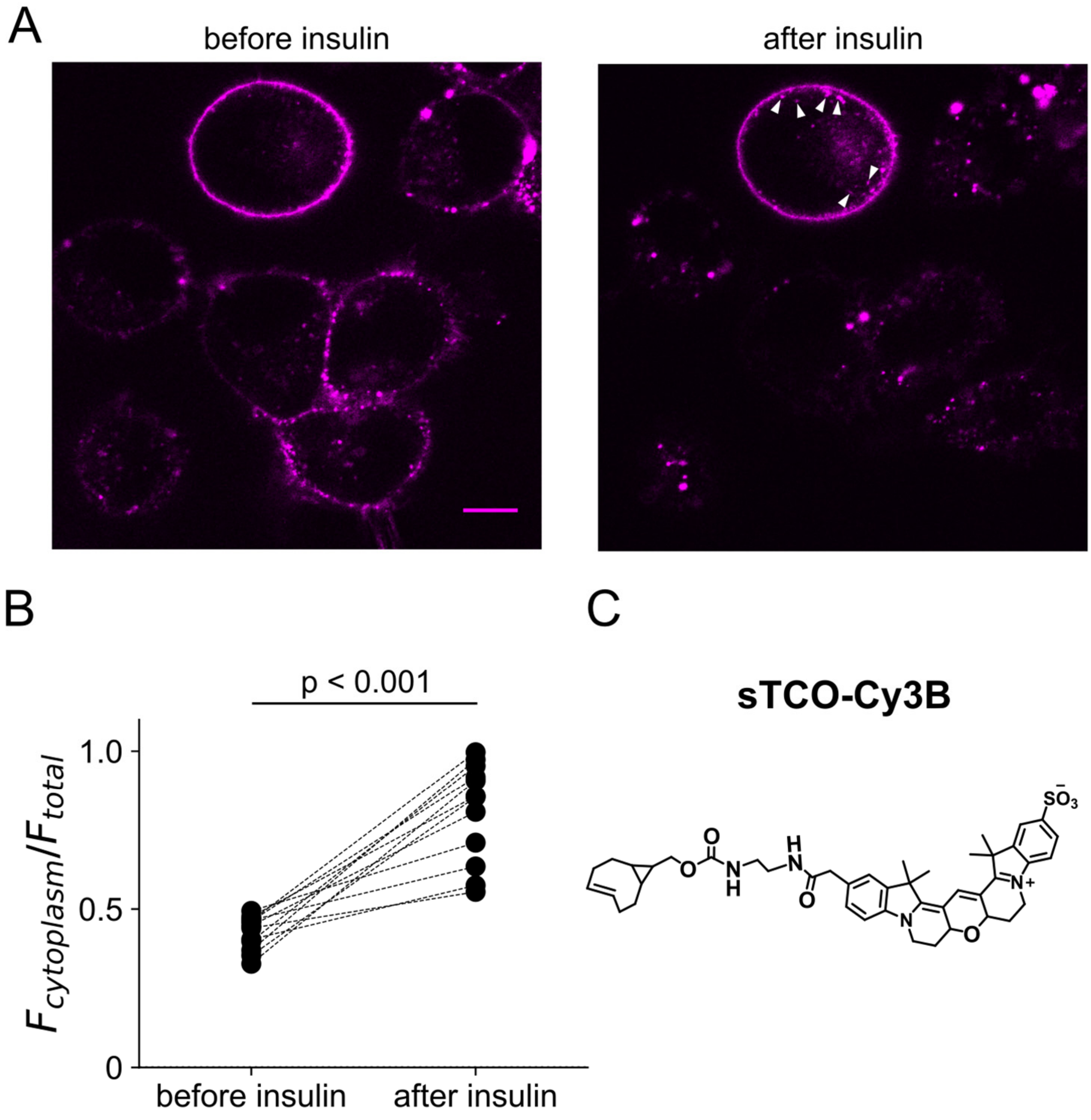

Figure 3-fig. supp. 2

bioRxiv preprint doi: https://doi.org/10.1101/2023.08.29.555449; this version posted May 14, 2024. The copyright holder for this preprint (which was not certified by peer review) is the author/funder, who has granted bioRxiv a license to display the preprint in perpetuity. It is made available under aCC-BY 4.0 International license.

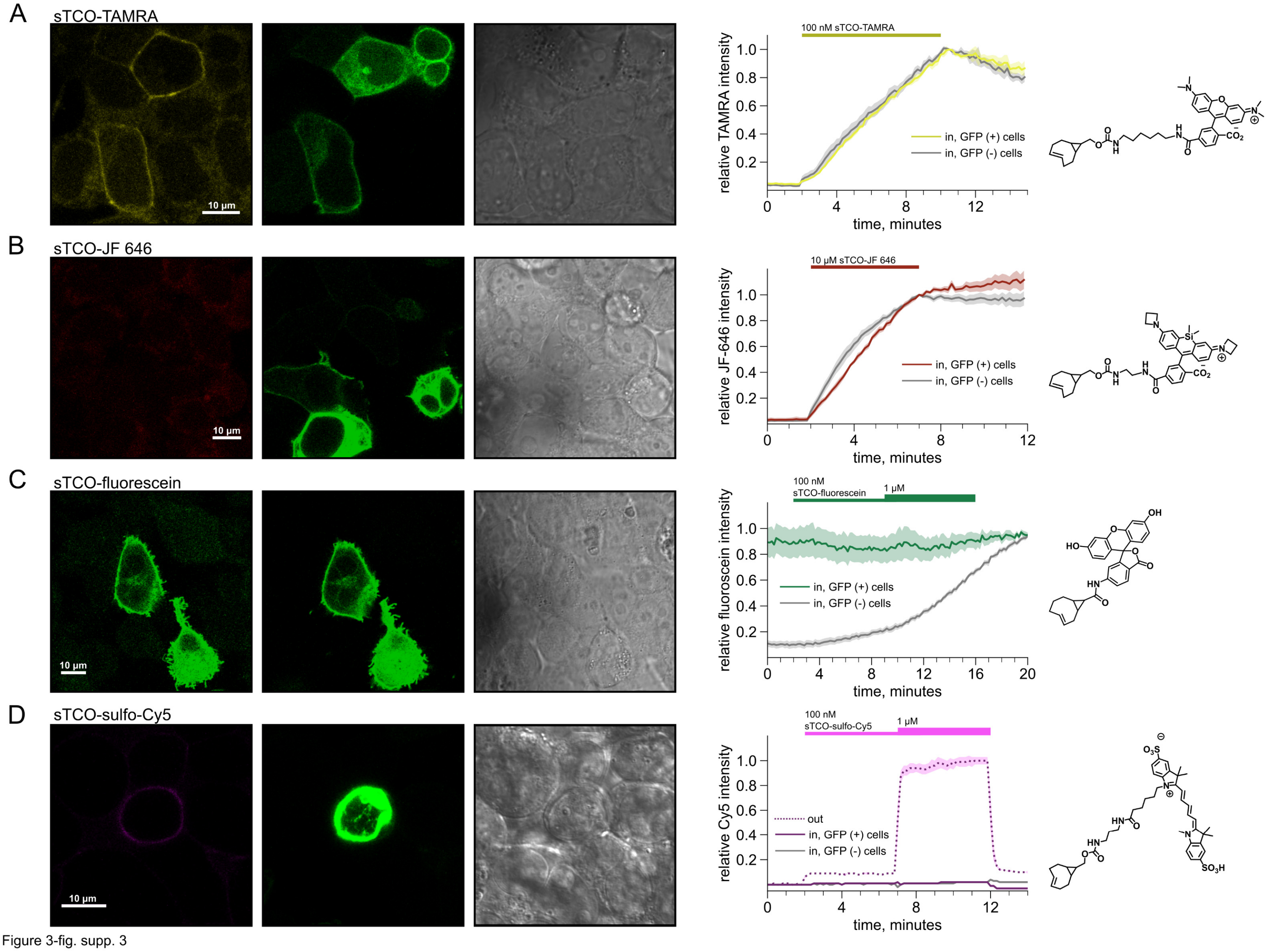

Figure 3-fig. supp. 3

bioRxiv preprint doi: https://doi.org/10.1101/2023.08.29.555449; this version posted May 14, 2024. The copyright holder for this preprint (which was not certified by peer review) is the author/funder, who has granted bioRxiv a license to display the preprint in perpetuity. It is made available under aCC-BY 4.0 International license.

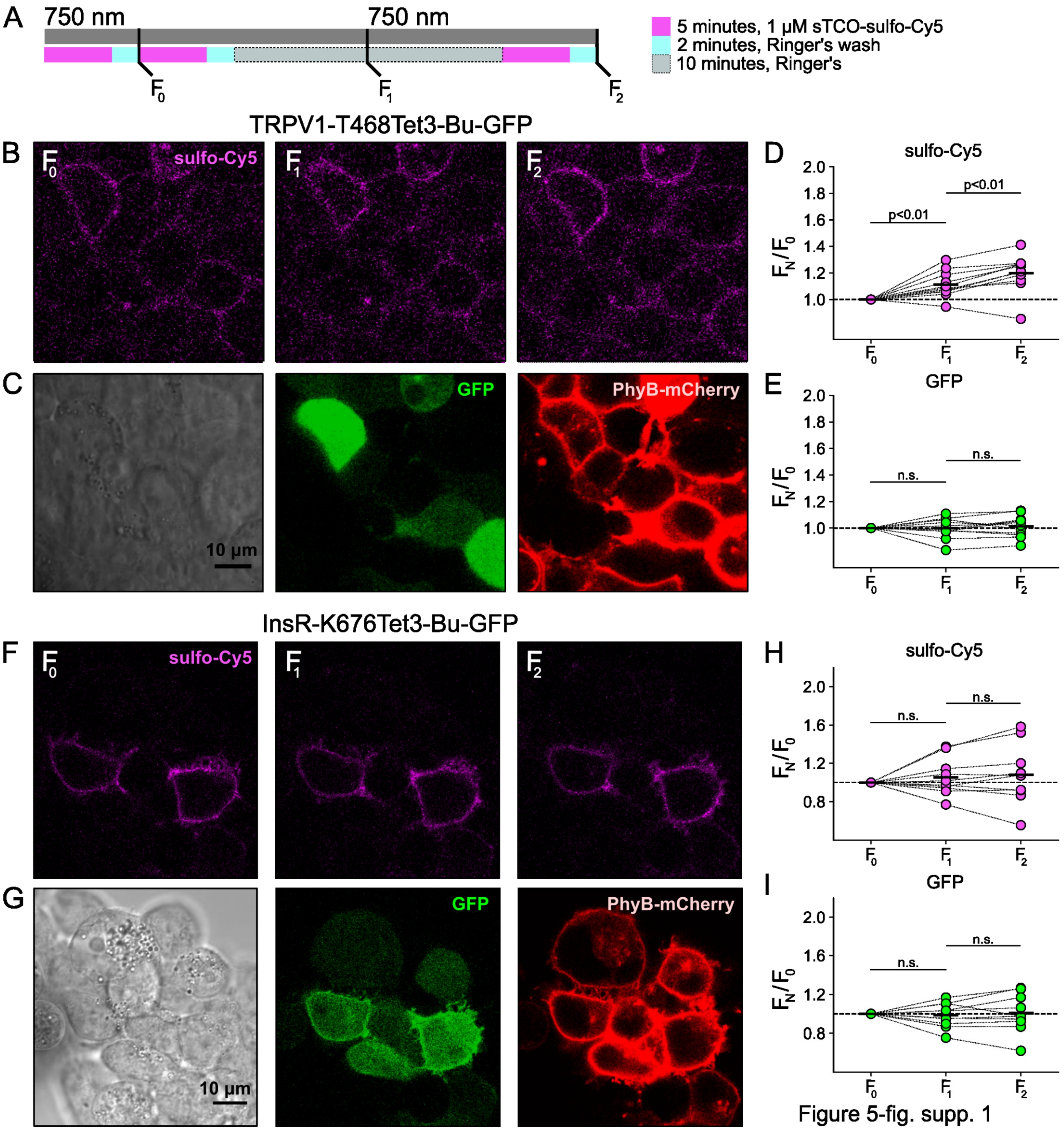

Figure 5-fig. supp. 1

bioRxiv preprint doi: https://doi.org/10.1101/2023.08.29.555449; this version posted May 14, 2024. The copyright holder for this preprint (which was not certified by peer review) is the author/funder, who has granted bioRxiv a license to display the preprint in perpetuity. It is made available under aCC-BY 4.0 International license.

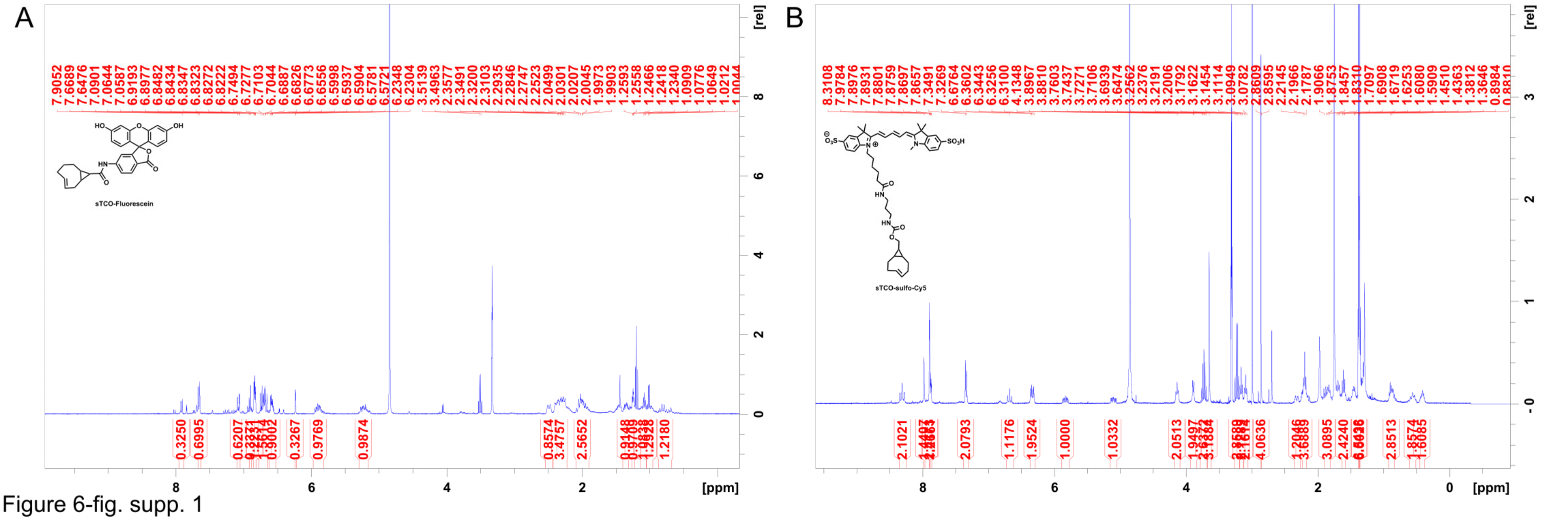

Figure 6-fig. supp. 1
