# Supplementary material for: Genetic code expansion, click chemistry, and light-activated PI3K reveal details of membrane protein trafficking downstream of receptor tyrosine kinases
Source: bioRxiv. 2024 May 1:2023.08.29.555449. Originally published 2023 Aug 30. Preprint. [Version 2] doi: 10.1101/2023.08.29.555449 (PMC10491195; doi:10.1101/2023.08.29.555449)
Supplement: Supplementary file 1 [file NIHPP2023.08.29.555449v2-supplement-1.pdf]

bioRxiv preprint doi: https://doi.org/10.1101/2023.08.29.555449; this version posted May 1, 2024. The copyright holder for this preprint (which was not certified by peer review) is the author/funder, who has granted bioRxiv a license to display the preprint in perpetuity. It is made available under aCC-BY 4.0 International license.

Figure 1 – figure supplement 1. PIF-YFP translocates to PM in response to 650 nm light and back to the cytosol in response to 750 nm light.

The PhyB –PIF light-inducible interaction is rapid (a few tens of seconds) and fully reversible (within ~30 seconds) and can be repeated multiple times. (A) Schematic diagram for optogenetic PhyB-PIF system. PhyB-mCherry loaded with the chromophore phycocyanobilin localizes to the PM due to a CAAX tag to induce lipidation (squiggly line). Illumination with 650 nm light induces a conformational change in PhyB that increases its affinity for PIF-YFP, effectively recruiting PIF-YFP to the PM. The conformational change in PhyB reverses with illumination with 750 nm light, causing PIF-YFP to dissociate and return to the cytoplasm. (B) A representative confocal experiment with an NIH3T3 cell stably expressing PhyB-mCherry-CAAX and PIF-YFP. Images were obtained at times indicated in C and are all shown on the same lookup table. During illumination with 650 nm light, PIF-YFP translocated to the PM quickly, with a corresponding decrease in cytoplasmic fluorescence. (C) Ratio of measured PIF fluorescence at region of interest (ROI) placed at the PM ($F_{Membrane}$) and cytoplasm ($F_{cytosol}$) from the cell in B. These data recapitulate previously published data (Levskaya et al., 2009; Toettcher et al., 2011).

Figure 1 – figure supplement 2. Detection of PI(3,4,5)P_3 generation by PIF-iSH2-YFP at the PM using GRP1-PH-CFP. (A) TIRF measurements of F-11 cells expressing PhyB-mCherry, PIF-iSH2-YFP, and GRP1-PH-CFP (n = 9). Upon 650 nm illumination, PIF PIF-iSH2-YFP translocated to the PM quickly (upper trace) while GRP1-PH-CFP moved slower (lower trace). Normalized to the initial 3 min control period. (B) Scatter plot of PIF-iSH2-YFP and GRP1-PH-CFP fluorescence for individual cells. Each point represents the 30 s average for 750 nm (2.5-3

bioRxiv preprint doi: https://doi.org/10.1101/2023.08.29.555449; this version posted May 1, 2024. The copyright holder for this preprint (which was not certified by peer review) is the author/funder, who has granted bioRxiv a license to display the preprint in perpetuity. It is made available under aCC-BY 4.0 International license.

min), 650 nm (4.5-5 min), and 750 nm (12.5-13 min). Translocation of both PIF-iSH2-YFP and GRP1-PH-CFP is reversible.

Figure 2 – figure supplement 1. 750 nm light fails to cause iSH2 dissociation from the PM in TRPV1-expressing cells.

(A) TIRF measurements of PIF-iSH2-YFP from F-11 cells expressing TRPV1-CFP in F-11 cells and HEK293T/17 cells, as indicated (n = 5 – 17). The color code and lines/envelopes have the same meaning as in Figure 2. Inset cartoons depict the model for retention of iSH2 at the PM via binding to TRPV1, which contains the PI3K-binding ARD domain. (B) Scatter plot of PIF-

bioRxiv preprint doi: https://doi.org/10.1101/2023.08.29.555449; this version posted May 1, 2024. The copyright holder for this preprint (which was not certified by peer review) is the author/funder, who has granted bioRxiv a license to display the preprint in perpetuity. It is made available under aCC-BY 4.0 International license.

iSH2-YFP fluorescence for individual cells. Each point represents the 20 s average for 750 nm (1.33 - 1.66 min), 650 nm (4.33 - 4.66 min), and 750 nm (7.33 - 7.66 min). Translocation of PIF-iSH2-YFP is irreversible. (C,D) The same experiments with continuous illumination at 750 nm (n = 5 – 13).

Figure 2 – figure supplement 2. figure supplement 3. PIF-YFP dissociates from the PM in response to 750 nm light even in TRPV1-expressing cells.

TRPV1 does not retain PIF lacking iSH2 at the PM of F-11 cells. (A) Normalized TIRF fluorescence was recorded in F-11 cells transfected with PhyB-mCherry-CAAX, PIF-YFP (no iSH2 domain), and either TRPV1-CFP or TRPV1 (no fluorescent tag). Reversible translocation of PIF-YFP upon 650 nm illumination demonstrates that PIF lacking iSH2 domain does not interact with TRPV1 channels on the PM (n = 8). (B) Scatter plot of PIF-iSH2-YFP fluorescence for individual cells. Each point represents the 20 s average for 750 nm (1.33 - 1.66 min), 650 nm (4.33 - 4.66 min), and 750 nm (7.33 - 7.66 min).

Figure 2 – figure supplement 3. 750 nm light succeeds in causing iSH2 dissociation from the PM in TRPM4-expressing cells.

(A) TIRF measurements of PIF-iSH2-YFP from F-11 and HEK293T/17 cells expressing TRPM4-CFP (n = 7 -17). Inset cartoons depict the model for no retention of iSH2 at the PM via binding to TRPM4, which has no ankyrin repeats. (B) Scatter plot of PIF-iSH2-YFP fluorescence for individual cells. Each point represents the 20 s average for 750 nm (1.33 - 1.66 min), 650 nm (4.33 - 4.66 min), and 750 nm (7.33 - 7.66 min).

bioRxiv preprint doi: https://doi.org/10.1101/2023.08.29.555449; this version posted May 1, 2024. The copyright holder for this preprint (which was not certified by peer review) is the author/funder, who has granted bioRxiv a license to display the preprint in perpetuity. It is made available under aCC-BY 4.0 International license.

Figure 3– figure supplement 1. Incorporation of Tet3-Bu into TRPV1 and InsR.

(A) Whole-cell patch clamp recording demonstrates that TRPV1 incorporating Tet3-Bu remains functional. Application of 1 μM capsaicin to HEK-293T/17 cells transfected with either TRPV1-GFP (top) or TRPV1-468Tet3-Bu (bottom) induced inward currents at a holding potential of -60 mV. (B) Collected whole-cell electrophysiology data from all cells with peak capsaicin-activated currents normalized to the area of cell membrane (n = 8 and 9 for TRPV1-GFP and TRPV1-468Tet3-Bu, respectively). (C) In-gel fluorescence screening demonstrates that expression of full-length TRPV1-468Tet3-Bu or InsR-676Tet3-Bu requires the presence of Tet3-Bu (‘Tet3 (+)’). Wild type TRPV1-GFP is shown for reference. Detergent-extracted cell lysates were run on SDS/PAGE. GFP fluorescence in the gels was measured and then cells were stained with Coomassie blue dye.

bioRxiv preprint doi: https://doi.org/10.1101/2023.08.29.555449; this version posted May 1, 2024. The copyright holder for this preprint (which was not certified by peer review) is the author/funder, who has granted bioRxiv a license to display the preprint in perpetuity. It is made available under aCC-BY 4.0 International license.

Figure 3– figure supplement 2. Activity of InsR-K676TAG measure as its recycling upon insulin treatment.

F-11 cells were transfected with the genetically modified receptor and inspected with a confocal microscope. (A) After labeling with membrane-impermeable sTCO-Cy3B dyes, the receptor was visible in the PM. Application of 100 nM insulin triggered the generation of endocytic granules (arrow heads). In some cells the receptor recycling was so vigorous that PM receptors nearly disappeared. (B) Scatter plot of the ratio of fluorescence between the cytoplasm and total cell area in individual cells (n = 12). (C) Structure of sTCO-Cy3B.

Figure 3– movie supplement 1. Activity of InsR-K676TAG measure as its recycling upon insulin treatment.

The same measurement as Figure 3– figure supplement 2A. Each image was obtained every 10 s. The time stamp is indicated on upper right corner and insulin application on upper left corner.

Figure 3 – figure supplement 3. Labelling of cells with sTCO conjugates of TAMRA, JF 646, fluorescein, and sulfo-Cy5.

HEK293T/17 cells expressing InsR-Tet3-Bu-GFP were labeled with sTCO dyes. For all dyes, shown are (left) confocal images of a representative field of HEK293T/17 cells incubated with the indicated concentration of sTCO-conjugated dye, (center) plots of the kinetics of cell labeling, and (right) a diagram of the fluorescent dye. The confocal images were obtained ~30 s after washing off of extracellular dyes. GFP and bright field images are presented to demonstrate GFP-expressing (GFP(+)) and non-expressing (GFP(-)) cells. (A) 100 nM sTCO-

bioRxiv preprint doi: https://doi.org/10.1101/2023.08.29.555449; this version posted May 1, 2024. The copyright holder for this preprint (which was not certified by peer review) is the author/funder, who has granted bioRxiv a license to display the preprint in perpetuity. It is made available under aCC-BY 4.0 International license.

TAMRA; (B) 10 µM sTCO-JF 646; (C) 100 nM and 1 µM sTCO-fluorescein; and (D) 100 nM and 1 µM sTCO-sulfo-Cy5. For the plots, the intracellular fluorescence was normalized to the peak value at the end of the dye applications (A-C) or to the fluorescence from 1 µM extracellular sTCO-sulfo-Cy5 (D). The thick lines represent the mean of 6 - 9 cells for each dye, and the envelopes represent the standard errors of the mean.

bioRxiv preprint doi: https://doi.org/10.1101/2023.08.29.555449; this version posted May 1, 2024. The copyright holder for this preprint (which was not certified by peer review) is the author/funder, who has granted bioRxiv a license to display the preprint in perpetuity. It is made available under aCC-BY 4.0 International license.

Figure 5 – figure supplement 1. Control experiment for light-activated PI3K-induced TRPV1 and InsR trafficking to the PM.

The same experiment as Figure 5 without 650 nm illumination. The cells were exposed to 750 nm throughout the recording (n = 11 and 10 for TRPV1-Tet3-Bu-GFP and InsR-676Tet3-Bu-GFP, respectively)

Figure 6 – figure supplement 1. ^{1}H NMR spectra of synthesized compounds (A) sTCO-fluorescein and (B) sTCO-sulfo-Cy5.

bioRxiv preprint doi: https://doi.org/10.1101/2023.08.29.555449; this version posted May 1, 2024. The copyright holder for this preprint (which was not certified by peer review) is the author/funder, who has granted bioRxiv a license to display the preprint in perpetuity. It is made available under aCC-BY 4.0 International license.

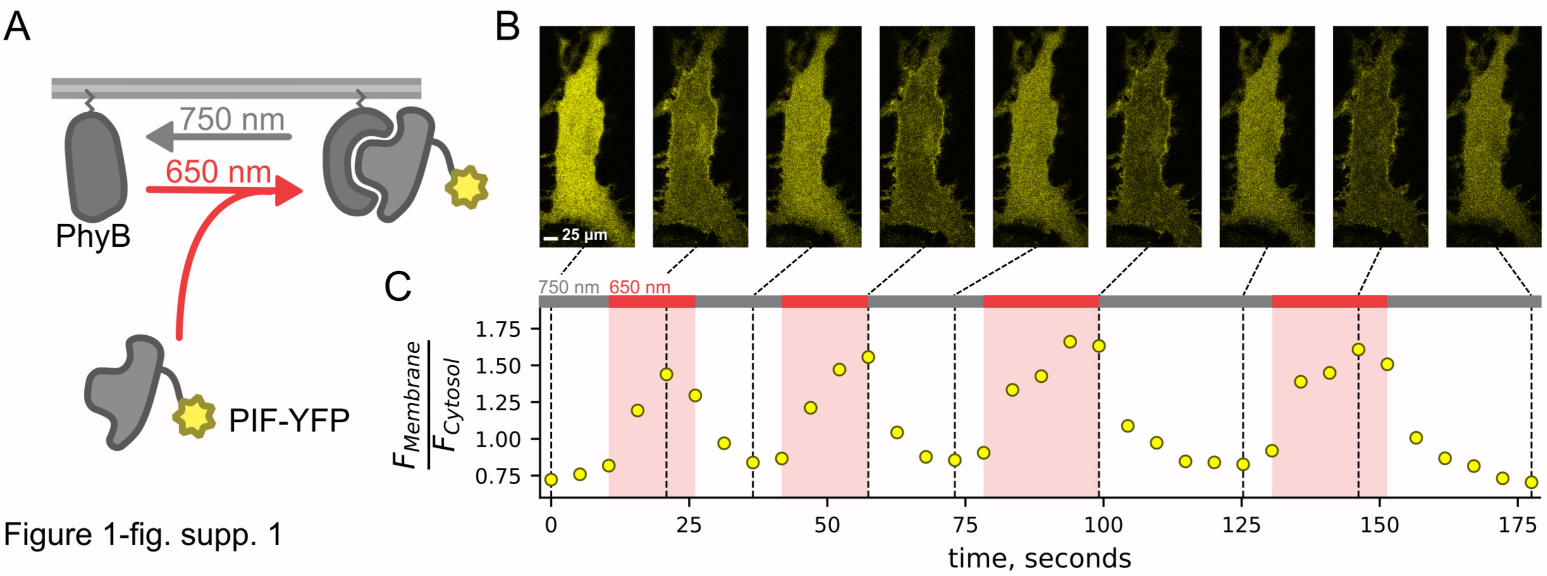

Figure 1-fig. supp. 1

bioRxiv preprint doi: https://doi.org/10.1101/2023.08.29.555449; this version posted May 1, 2024. The copyright holder for this preprint (which was not certified by peer review) is the author/funder, who has granted bioRxiv a license to display the preprint in perpetuity. It is made available under aCC-BY 4.0 International license.

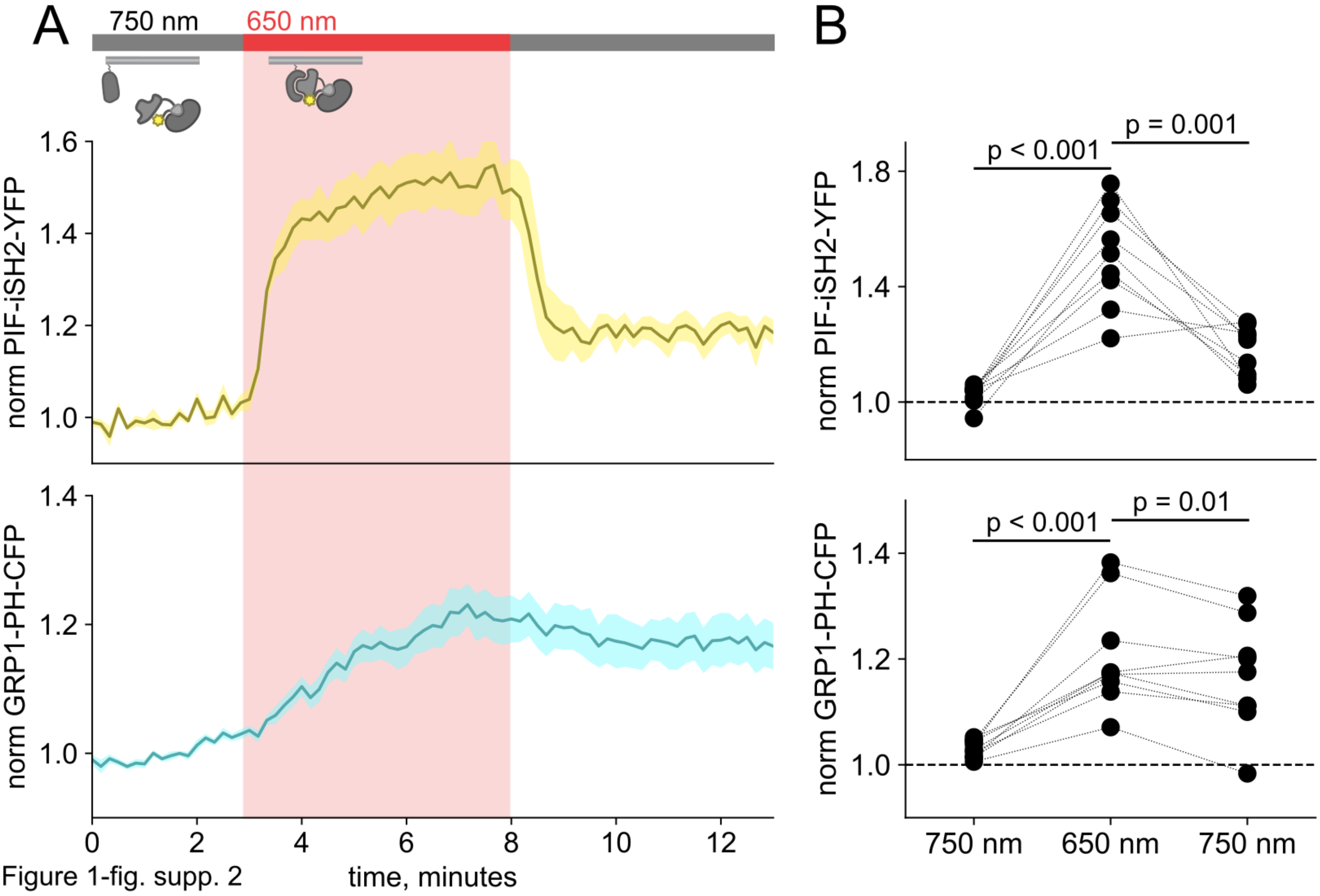

Figure 1-fig. supp. 2

bioRxiv preprint doi: https://doi.org/10.1101/2023.08.29.555449; this version posted May 1, 2024. The copyright holder for this preprint (which was not certified by peer review) is the author/funder, who has granted bioRxiv a license to display the preprint in perpetuity. It is made available under aCC-BY 4.0 International license.

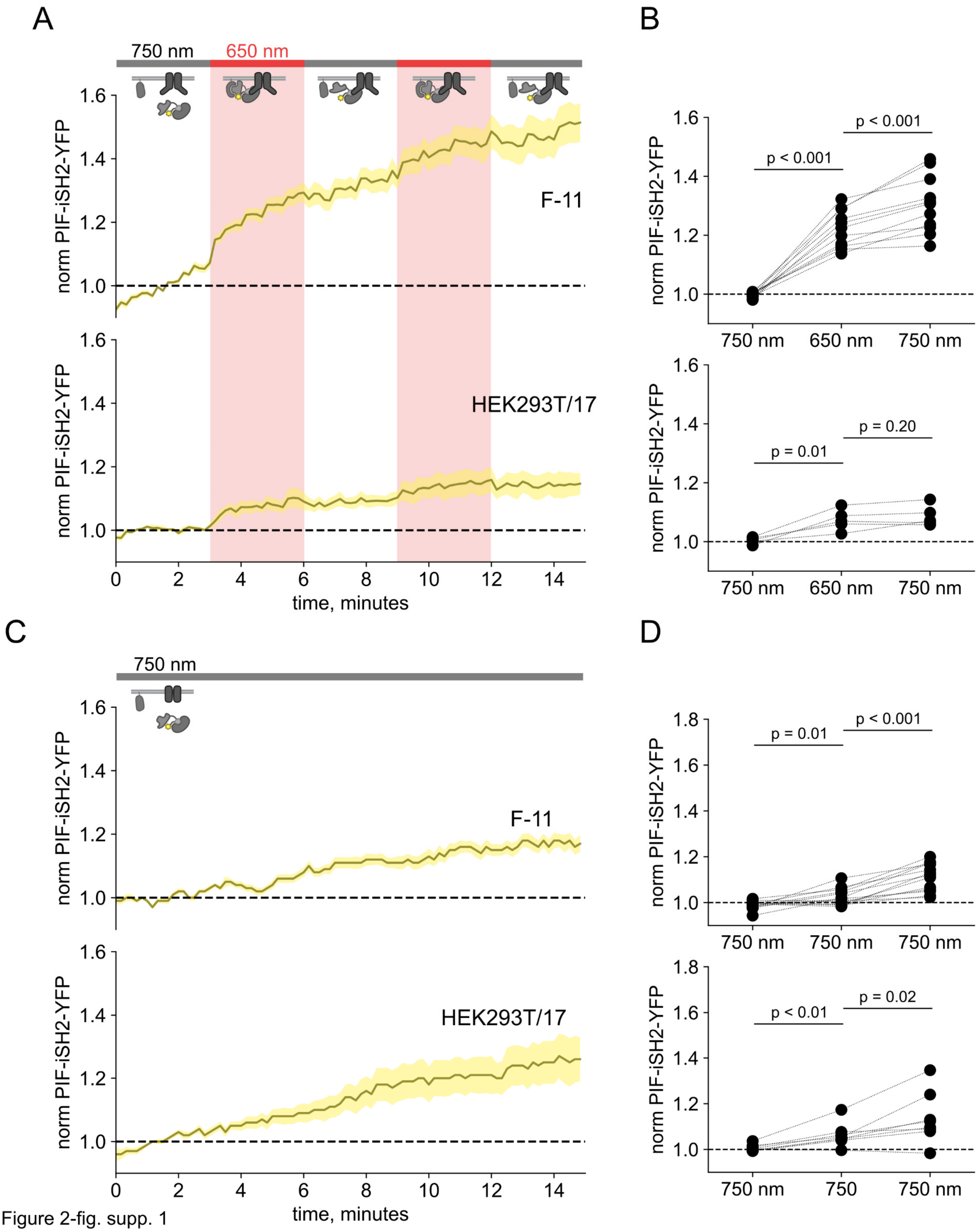

Figure 2-fig. supp. 1

bioRxiv preprint doi: https://doi.org/10.1101/2023.08.29.555449; this version posted May 1, 2024. The copyright holder for this preprint (which was not certified by peer review) is the author/funder, who has granted bioRxiv a license to display the preprint in perpetuity. It is made available under aCC-BY 4.0 International license.

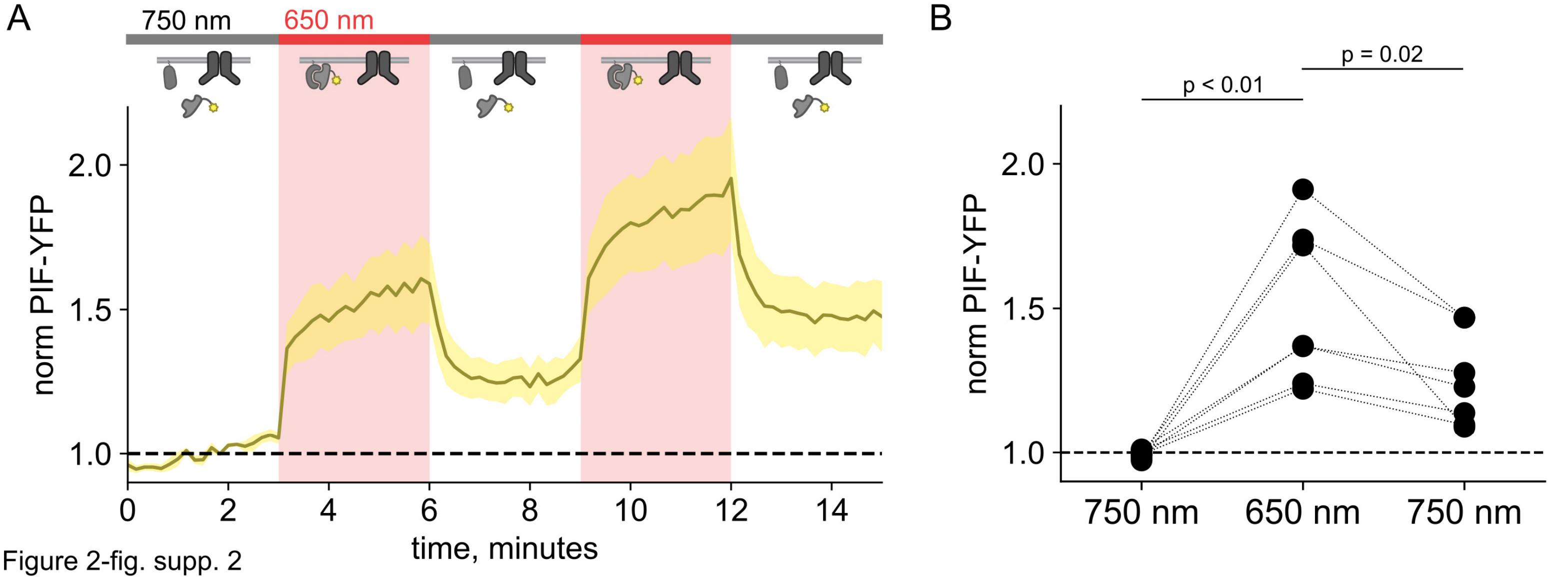

Figure 2-fig. supp. 2

bioRxiv preprint doi: https://doi.org/10.1101/2023.08.29.555449; this version posted May 1, 2024. The copyright holder for this preprint (which was not certified by peer review) is the author/funder, who has granted bioRxiv a license to display the preprint in perpetuity. It is made available under aCC-BY 4.0 International license.

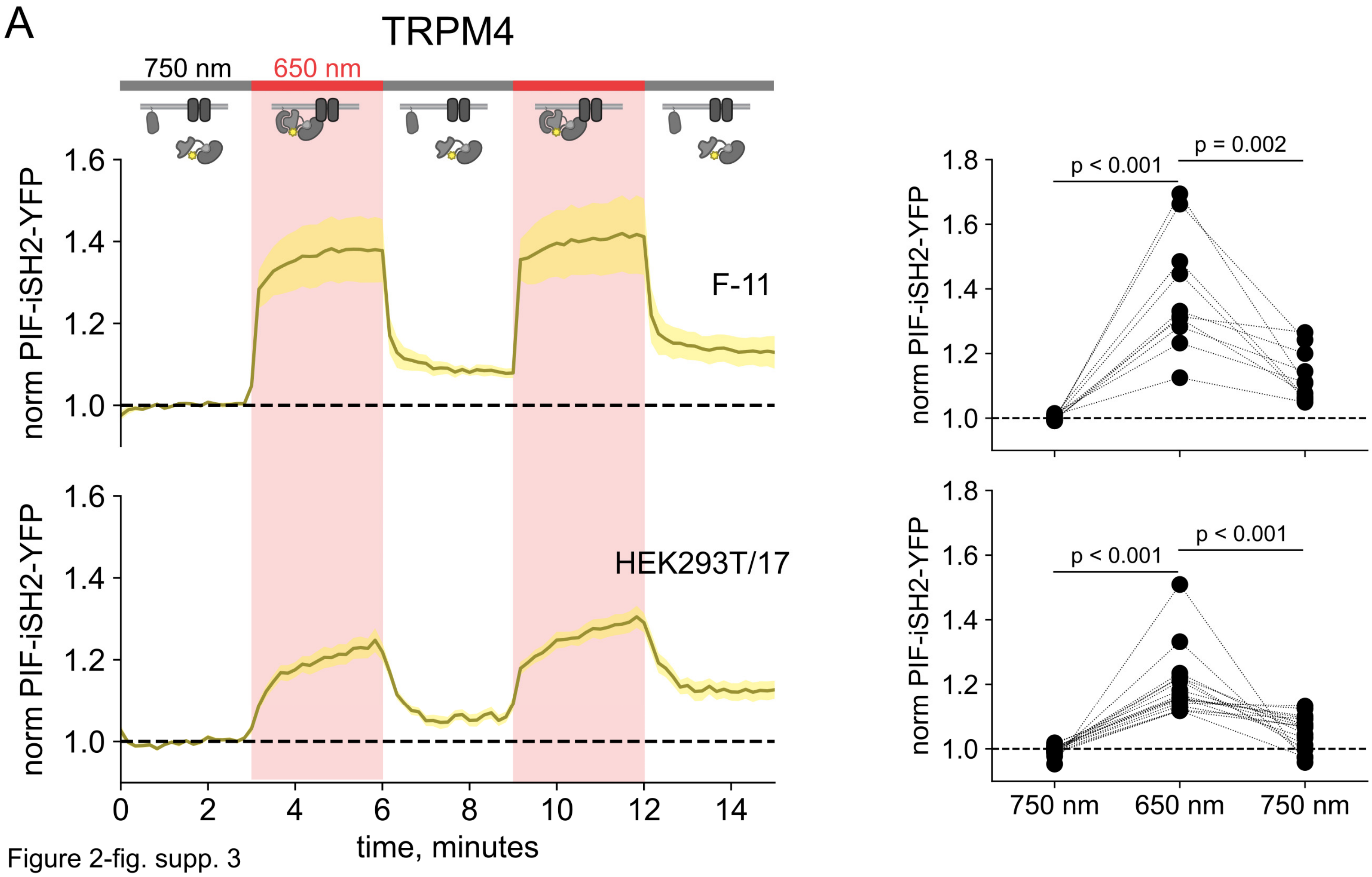

Figure 2-fig. supp. 3

bioRxiv preprint doi: https://doi.org/10.1101/2023.08.29.555449; this version posted May 1, 2024. The copyright holder for this preprint (which was not certified by peer review) is the author/funder, who has granted bioRxiv a license to display the preprint in perpetuity. It is made available under aCC-BY 4.0 International license.

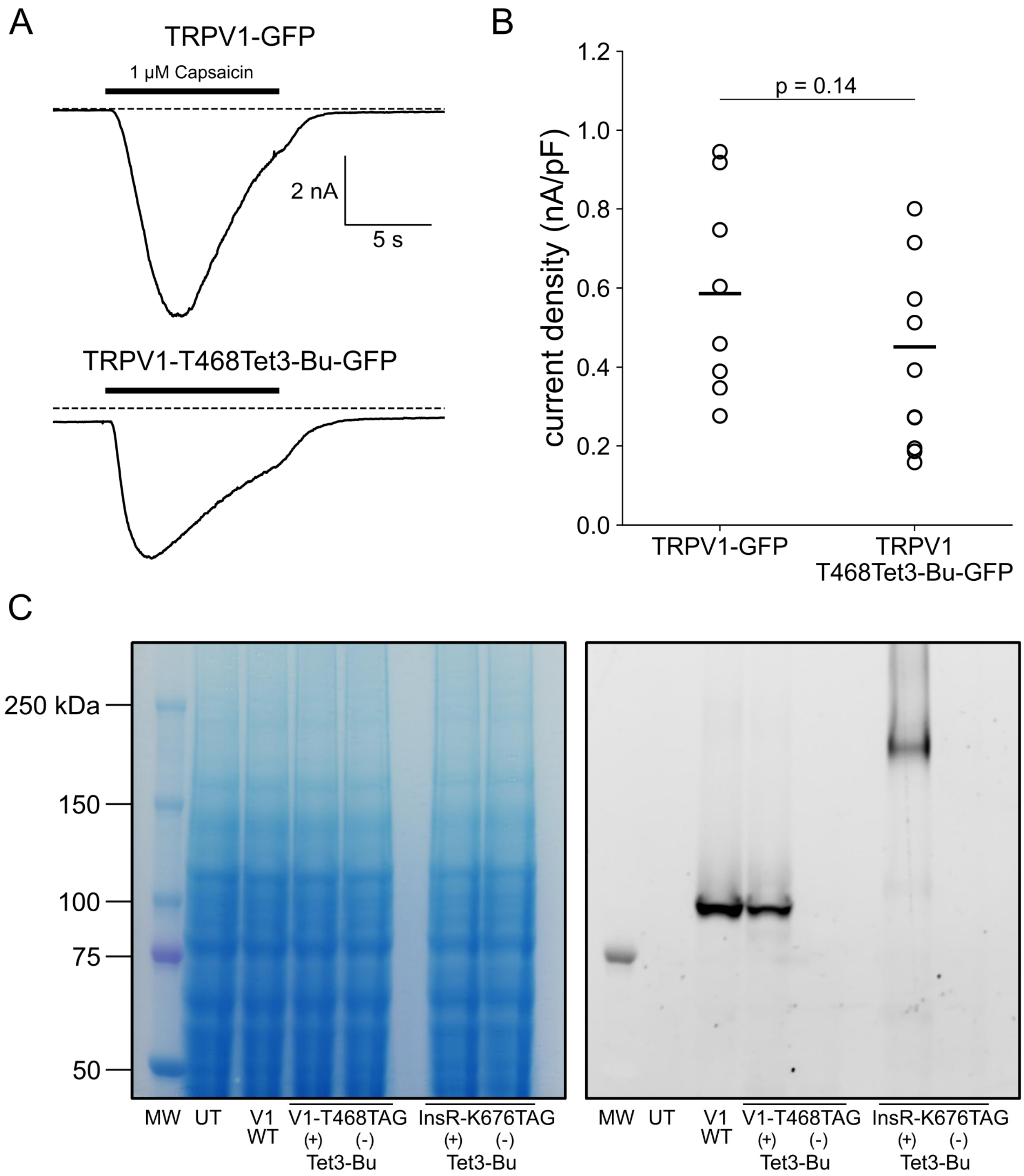

Figure 3-fig. supp. 1

bioRxiv preprint doi: https://doi.org/10.1101/2023.08.29.555449; this version posted May 1, 2024. The copyright holder for this preprint (which was not certified by peer review) is the author/funder, who has granted bioRxiv a license to display the preprint in perpetuity. It is made available under aCC-BY 4.0 International license.

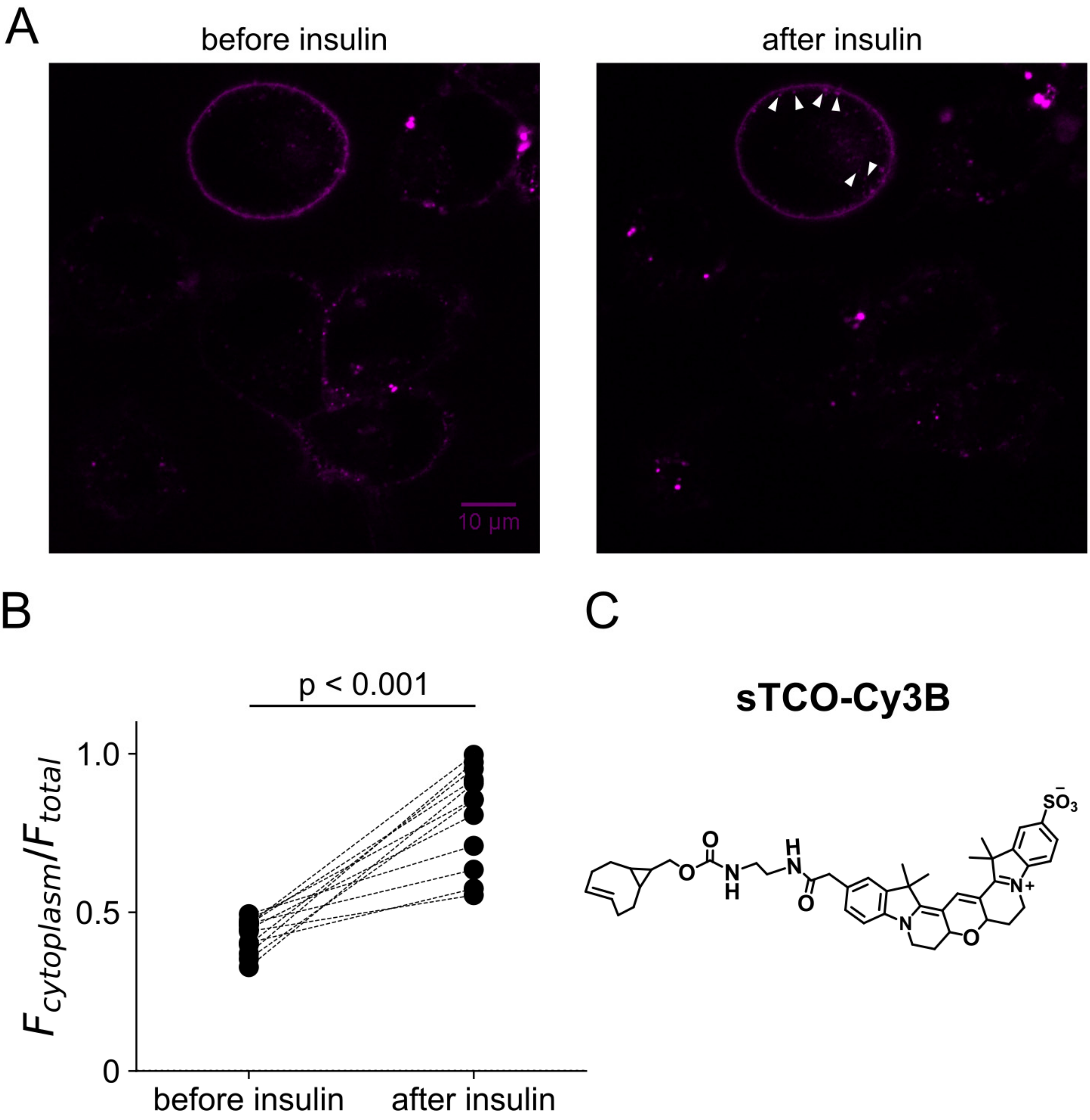

Figure 3-fig. supp. 2

bioRxiv preprint doi: https://doi.org/10.1101/2023.08.29.555449; this version posted May 1, 2024. The copyright holder for this preprint (which was not certified by peer review) is the author/funder, who has granted bioRxiv a license to display the preprint in perpetuity. It is made available under aCC-BY 4.0 International license.

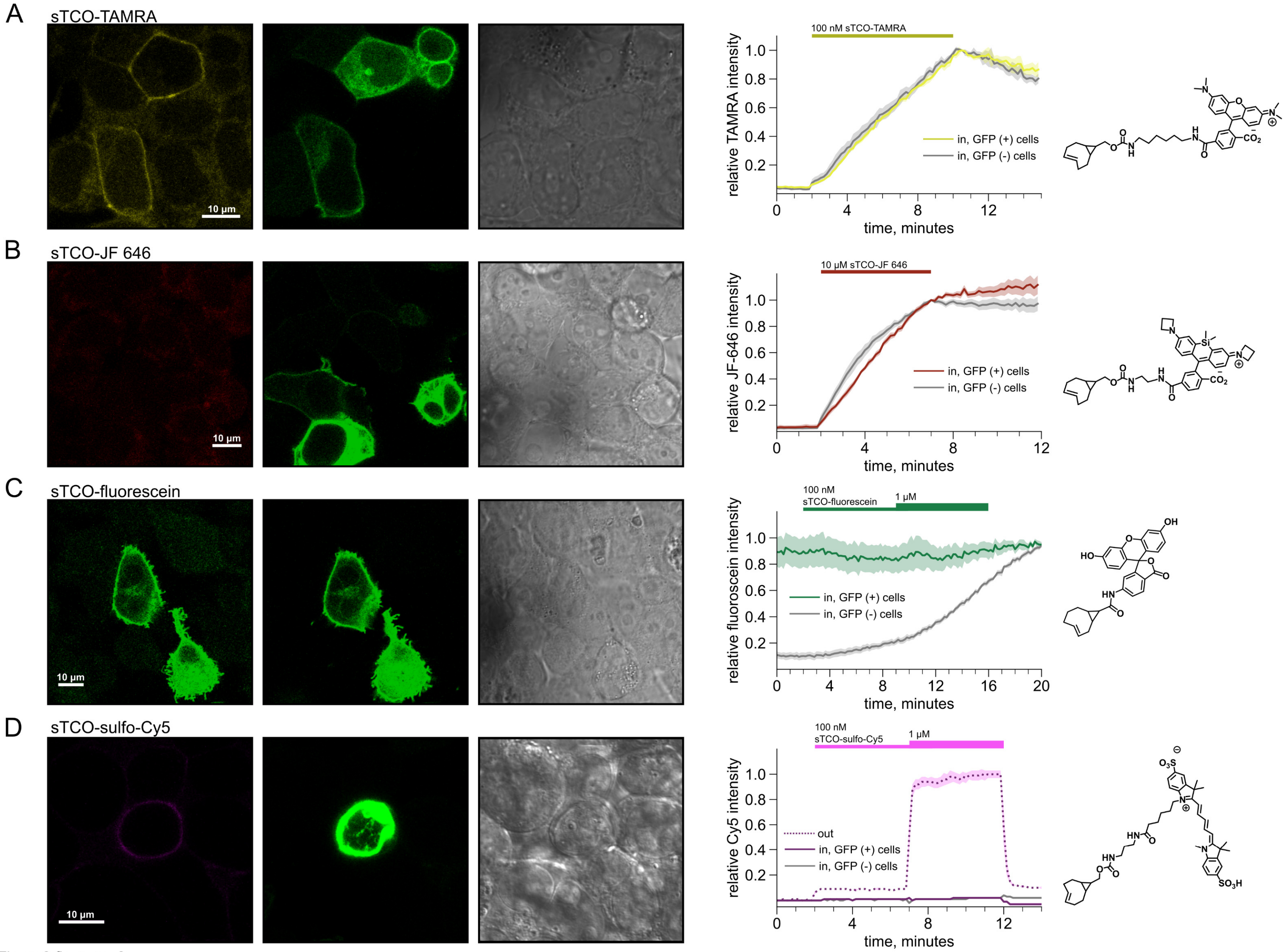

Figure 3-fig. supp. 3

bioRxiv preprint doi: https://doi.org/10.1101/2023.08.29.555449; this version posted May 1, 2024. The copyright holder for this preprint (which was not certified by peer review) is the author/funder, who has granted bioRxiv a license to display the preprint in perpetuity. It is made available under aCC-BY 4.0 International license.

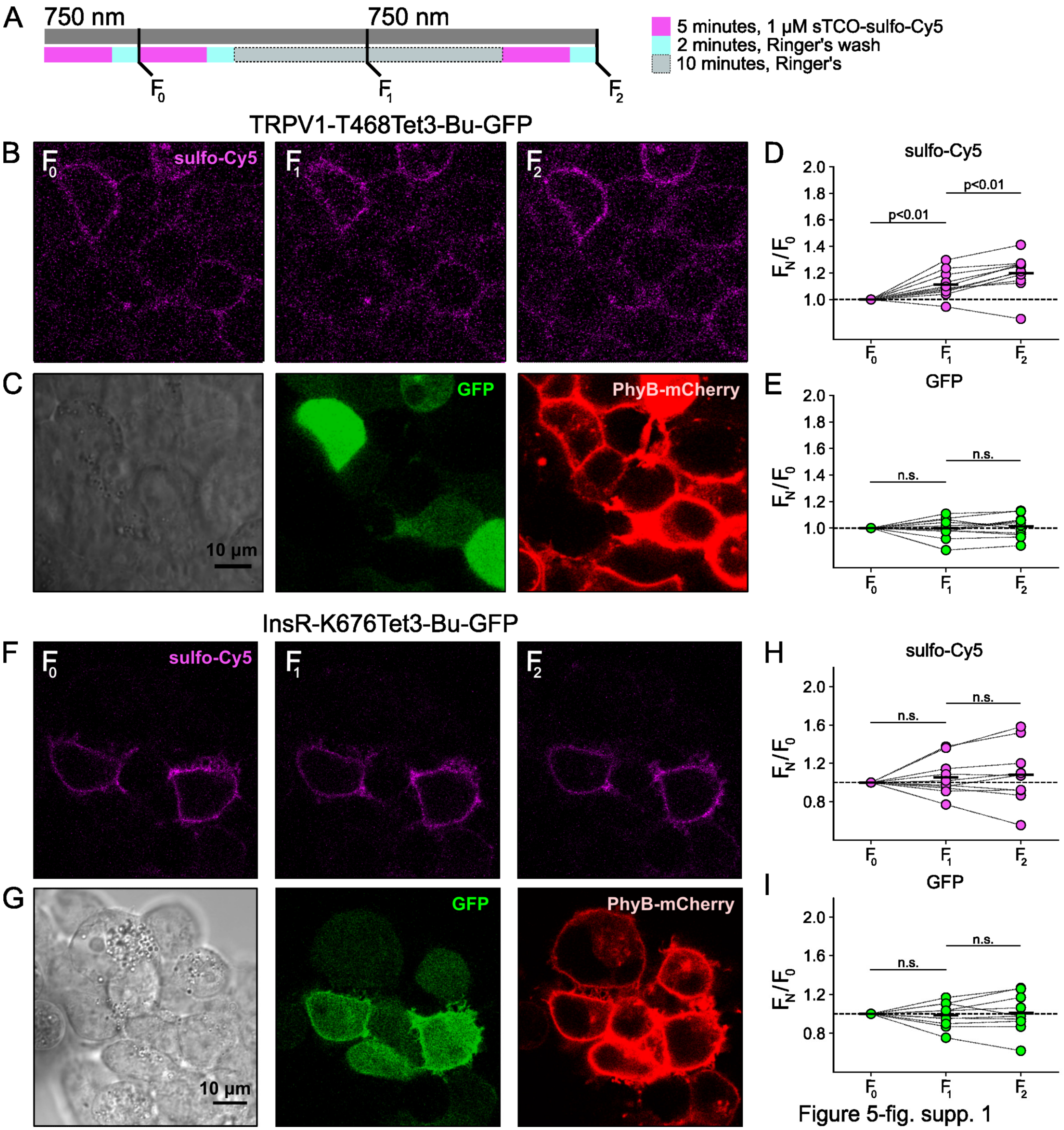

Figure 5-fig. supp. 1

bioRxiv preprint doi: https://doi.org/10.1101/2023.08.29.555449; this version posted May 1, 2024. The copyright holder for this preprint (which was not certified by peer review) is the author/funder, who has granted bioRxiv a license to display the preprint in perpetuity. It is made available under aCC-BY 4.0 International license.

A

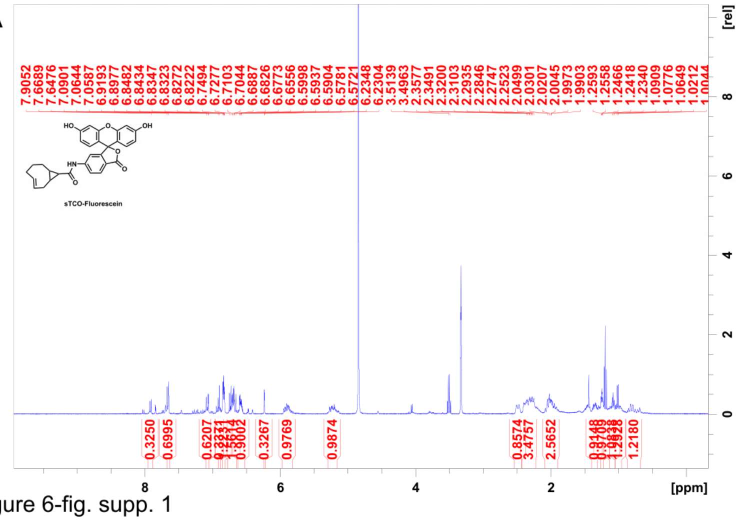

B

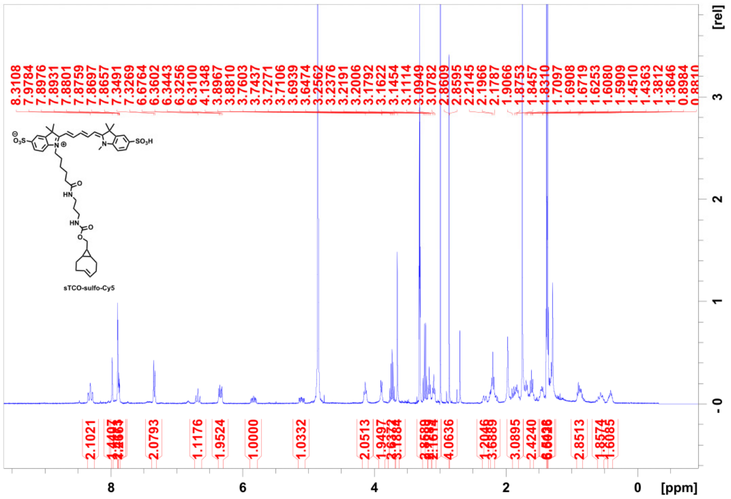

Figure 6-fig. supp. 1
