# Supplementary material for: OptoPI3K, genetic code expansion, and click chemistry reveal mechanisms underlying reciprocal regulation between TRPV1 and PI3K
Source: bioRxiv. 2023 Aug 30:2023.08.29.555449. Preprint. [Version 1] doi: 10.1101/2023.08.29.555449 (PMC10491195; doi:10.1101/2023.08.29.555449)
Supplement: Supplementary file 1 [file NIHPP2023.08.29.555449v1-supplement-1.pdf]

bioRxiv preprint doi: https://doi.org/10.1101/2023.08.29.555449; this version posted August 30, 2023. The copyright holder for this preprint (which was not certified by peer review) is the author/funder, who has granted bioRxiv a license to display the preprint in perpetuity. It is made available under aCC-BY 4.0 International license.

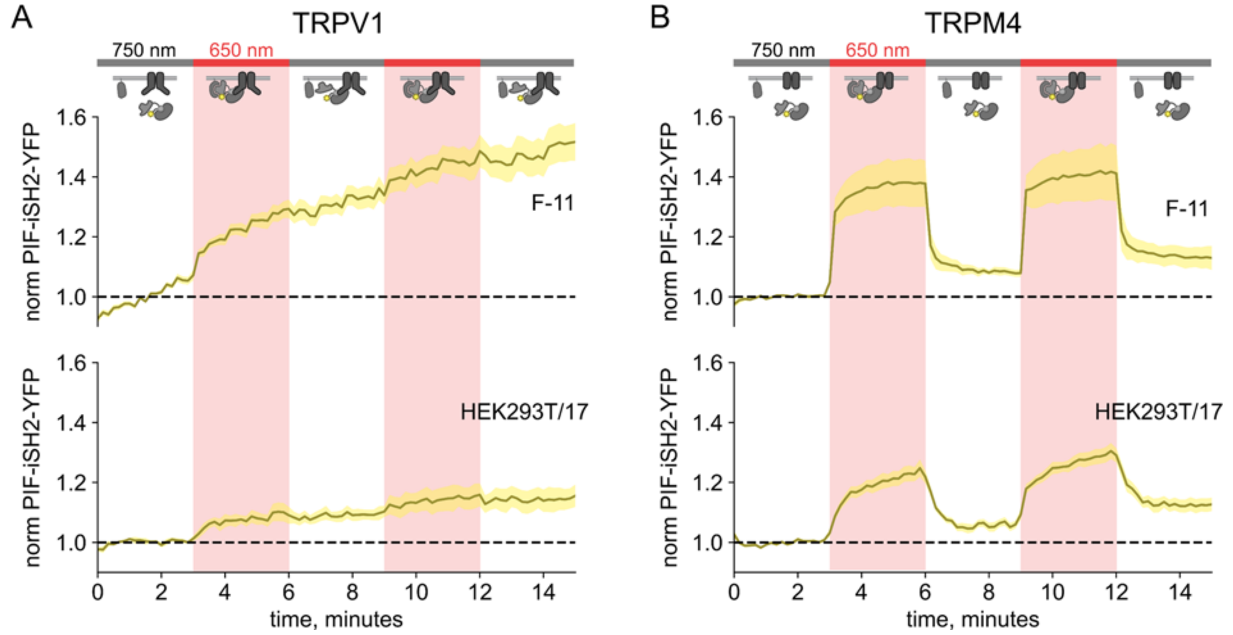

Figure 3 – figure supplement 1. TRPV1 traps PIF-iSH2-YFP at the PM, whereas TRPM4 does not.

bioRxiv preprint doi: https://doi.org/10.1101/2023.08.29.555449; this version posted August 30, 2023. The copyright holder for this preprint (which was not certified by peer review) is the author/funder, who has granted bioRxiv a license to display the preprint in perpetuity. It is made available under aCC-BY 4.0 International license.

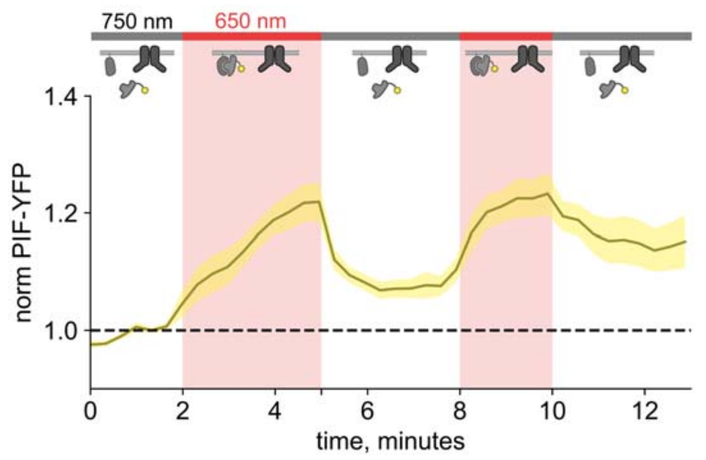

Figure 3 – figure supplement 2. PIF-YFP without iSH2 is not trapped at the PM by TRPV1.

bioRxiv preprint doi: https://doi.org/10.1101/2023.08.29.555449; this version posted August 30, 2023. The copyright holder for this preprint (which was not certified by peer review) is the author/funder, who has granted bioRxiv a license to display the preprint in perpetuity. It is made available under aCC-BY 4.0 International license.

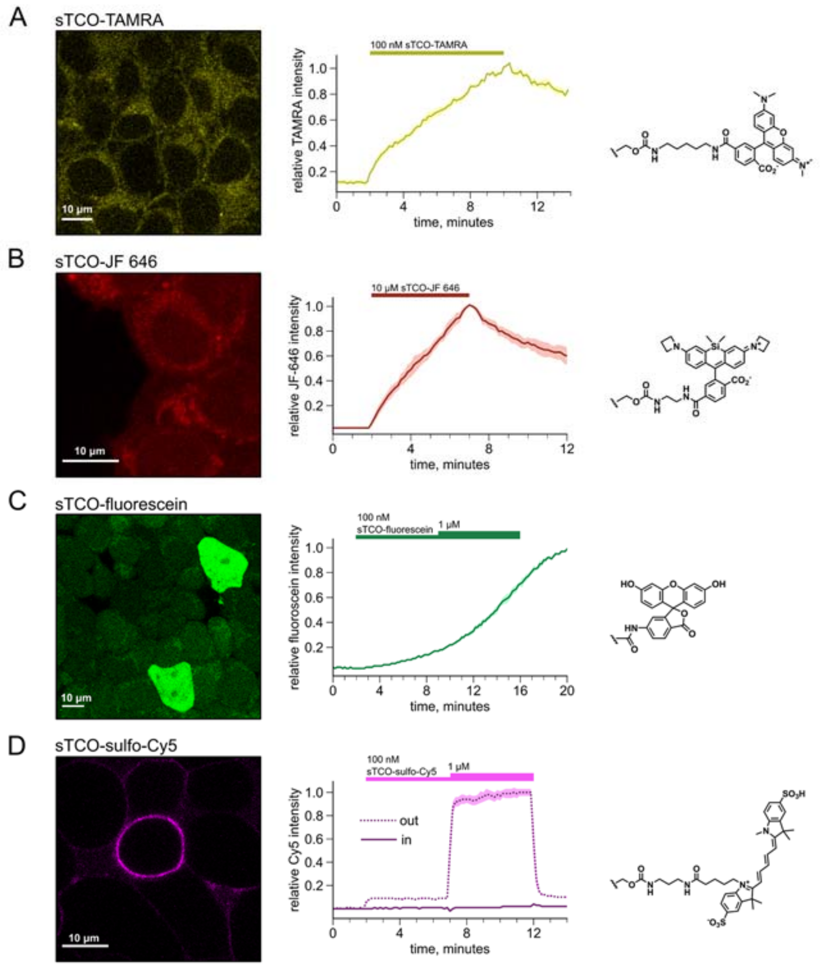

Figure 4– figure supplement 1. Labelling of cells with sTCO conjugates of TAMRA, JF 646, fluorescein, and sulfo-Cy5.

bioRxiv preprint doi: https://doi.org/10.1101/2023.08.29.555449; this version posted August 30, 2023. The copyright holder for this preprint (which was not certified by peer review) is the author/funder, who has granted bioRxiv a license to display the preprint in perpetuity. It is made available under aCC-BY 4.0 International license.

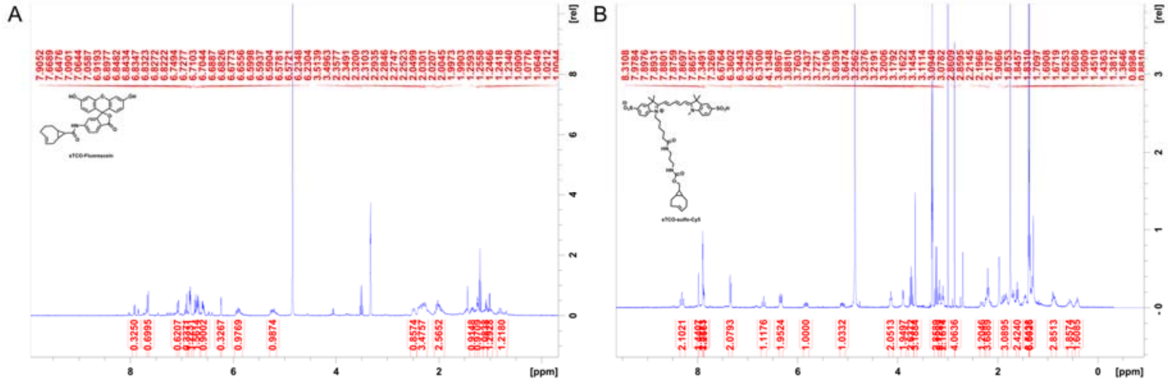

Figure 9 – figure supplement 1. ^{1}H NMR spectra of synthesized compounds

bioRxiv preprint doi: https://doi.org/10.1101/2023.08.29.555449; this version posted August 30, 2023. The copyright holder for this preprint (which was not certified by peer review) is the author/funder, who has granted bioRxiv a license to display the preprint in perpetuity. It is made available under aCC-BY 4.0 International license.

Figure 3 – figure supplement 1. TRPV1 traps iSH2 at the PM but TRPM4 does not.

TIRF measurements of PIF-iSH2-YFP from F-11 cells expressing (A) TRPV1-YFP or (B) TRPM4-YFP, for F-11 cells and HEK293T/17 cells, as indicated (n = 5 – 17). The color code and lines/envelopes have the same meaning as in Figure 3. Inset cartoons depict the model for retention of iSH2 at the PM via binding to TRPV1, which contains the PI3K-binding ARD domain, but not by TRPM4, which has no ankyrin repeats.

Figure 3 – figure supplement 2 PIF-YFP without iSH2 is not trapped at the PM by TRPV1.

TRPV1 does not retain PIF lacking iSH2 at the PM of F-11 cells. Normalized TIRF fluorescence was recorded in F-11 cells transfected with PhyB-mCherry-CAAX, PIF-YFP (no iSH2 domain), and either TRPV1-CFP or TRPV1 (no fluorescent tag). Reversible translocation of PIF-YFP upon 650 nm

bioRxiv preprint doi: https://doi.org/10.1101/2023.08.29.555449; this version posted August 30, 2023. The copyright holder for this preprint (which was not certified by peer review) is the author/funder, who has granted bioRxiv a license to display the preprint in perpetuity. It is made available under aCC-BY 4.0 International license.

illumination demonstrates that PIF lacking ISH2 domain does not interact with TRPV1 channels on the PM. The black trace represents the mean and the yellow envelope represents the standard error of the mean (n = 8).

Figure 4 – figure supplement 1. Labelling of cells with sTCO conjugates of TAMRA, JF 646, fluorescein, and sulfo-Cy5.

For all dyes, shown are (left) confocal images of a representative field of HEK293T/17 cells incubated with the indicated concentration of sTCO-conjugated dye, (center) plots of the kinetics of cell labeling, and (right) a diagram of the fluorescent dye. The images were obtained ~30 s after washing off of extracellular dyes. (A) 100 nM sTCO-TAMRA; (B) 100 nM and 1 μM sTCO-fluorescein; (C) 10 μM sTCO-JF 646; and (D) 200 nM sTCO-sulfo-Cy5. For the plots, the intracellular fluorescence was normalized to (A-C) the

bioRxiv preprint doi: https://doi.org/10.1101/2023.08.29.555449; this version posted August 30, 2023. The copyright holder for this preprint (which was not certified by peer review) is the author/funder, who has granted bioRxiv a license to display the preprint in perpetuity. It is made available under aCC-BY 4.0 International license.

peak value at the end of the dye applications or (D) to the fluorescence from 1 uM extracellular sTOC-sulfo-Cy5 (G). The black traces represent the mean of 3-8 cells for each dye, and the envelopes represent the standard errors of the mean.

bioRxiv preprint doi: https://doi.org/10.1101/2023.08.29.555449; this version posted August 30, 2023. The copyright holder for this preprint (which was not certified by peer review) is the author/funder, who has granted bioRxiv a license to display the preprint in perpetuity. It is made available under aCC-BY 4.0 International license.

Figure 9 – figure supplement 1. ^{1}H NMR spectra of synthesized compounds (A) sTCO-fluorescein and (B) sTCO-sulfo-Cy5.
